# Benchmark study comparing liftover tools for genome conversion of epigenome sequencing data

**DOI:** 10.1093/nargab/lqaa054

**Published:** 2020-08-06

**Authors:** Phuc-Loi Luu, Phuc-Thinh Ong, Thanh-Phuoc Dinh, Susan J Clark

**Affiliations:** Epigenetics Research Laboratory, Genomics and Epigenetics Division, Garvan Institute of Medical Research, Sydney 2010, New South Wales, Australia; St Vincent's Clinical School, UNSW, Sydney 2010, New South Wales, Australia; Faculty of Public Health, University of Medicine and Pharmacy at Ho Chi Minh city, Ho Chi Minh city 70000, Vietnam; Department of Biotechnology, Nong Lam University, Ho Chi Minh city 70000, Vietnam; Epigenetics Research Laboratory, Genomics and Epigenetics Division, Garvan Institute of Medical Research, Sydney 2010, New South Wales, Australia; St Vincent's Clinical School, UNSW, Sydney 2010, New South Wales, Australia

## Abstract

As reference genome assemblies are updated there is a need to convert epigenome sequence data from older genome assemblies to newer versions, to facilitate data integration and visualization on the same coordinate system. Conversion can be done by re-alignment of the original sequence data to the new assembly or by converting the coordinates of the data between assemblies using a mapping file, an approach referred to as ‘liftover’. Compared to re-alignment approaches, liftover is a more rapid and cost-effective solution. Here, we benchmark six liftover tools commonly used for conversion between genome assemblies by coordinates, including *UCSC liftOver*, *rtracklayer::liftOver*, *CrossMap*, *NCBI Remap*, *flo* and *segment_liftover* to determine how they performed for whole genome bisulphite sequencing (WGBS) and ChIP-seq data. Our results show high correlation between the six tools for conversion of 43 WGBS paired samples. For the chromatin sequencing data we found from interval conversion of 366 ChIP-Seq datasets, *segment_liftover* generates more reliable results than *USCS liftOver*. However, we found some regions do not always remain the same after liftover. To further increase the accuracy of liftover and avoid misleading results, we developed a three-step guideline that removes aberrant regions to ensure more robust genome conversion between reference assemblies.

## INTRODUCTION

The first commercial next-generation sequencing platform was successfully developed in 2005, which allowed millions of short-reads to be generated concurrently (1). Rather than mapping these short-reads to each other, which is known as *de novo* assembling, mapping to a reference genome is more efficient and time-effective. However, achieving a comprehensive reference assembly is challenging. Since its first edition was published in 2001 (2), the human reference genome has been updated regularly (Supplementary Table S1). The most commonly used genome assemblies in biomedical science are GRCh37 (hg19) and GRCh38 (hg38), released in 2006 and 2013, respectively. Generally, for each new genome assembly release remapping is recommended due to its updated features. Thus, methods to convert between assemblies are essential to achieve concordant datasets that allow meta-analysis or comparison studies. Importantly the GRC (Genome Reference Consortium) will review data for the next human assembly update, which emphasizes the ongoing need for further genome conversions.

Epigenome sequencing datasets also require remapping to the newer genome assemblies, however a comprehensive benchmarking comparison of tools that can be used to convert between assemblies for DNA methylation or chromatin genome-wide sequencing data has not yet been explored. Among DNA methylation analyses, whole genome bisulphite sequencing (WGBS) is acknowledged as the gold standard and is increasingly used in methylome studies (3). However, as WGBS can profile all CpGs (∼28 million) in humans (4), the methylome of one sample can generate up to 1 TB of data at 30× coverage and take 2–3 days using 16 cores of CPU to analyze (3). Several publicly available pipelines have been created to process the WGBS from raw reads to calling DNA methylation, such as *methylKit* (5), *BS-Seeker3* (6), *Bismark* (7), *MethPipe* (8), *P3Bsseq* (9) and *Meth10X* (3). The most widely used technique to detect histone modification patterns is Chromatin ImmunoPrecipitation Sequencing (ChIP-Seq) technology (10). In addition, ChIP-Seq can also profile transcription factor binding sites (TFBS). A ChIP-Seq full process typically generates 10–15 GB of data for a single sample, and takes 6–8 h using eight cores of CPU to analyze. Genome and epigenome analyses also utilize published data from different public sources, such as TGCA, GEO-NCBI, 1000 Genomes Project, ENCODE Project, FANTOM Project, Epigenome Roadmap, IHEC, BLUEPRINT and therefore an important task is to convert the different sequencing data into a consistent genomic coordinate system, such as hg38.

The best approach is to re-align the raw sequence in FASTA format to the new reference build. However, this method is computationally expensive and the raw sequence data are not always accessible because of the massive volume and potential ethical issues around human data. An alternative approach is to convert the genome data between assemblies without realignment. Among the methods, liftover tools are more favored due to their simplicity and versatility. The tools are called liftover because they ‘lift’ genome positions (also called as coordinates) in a reference assembly ‘over’ to another genome build. Genomic liftover only requires storage-friendly and tabulated files that contain coordinates data, which are ready for downstream analysis. The liftover technique can be applied in any situation that requires conversion between different versions of the reference genome. Liftover is also used to perform cross species mapping, which takes coordinates of genes in one species to identify the corresponding coordinates in other species (11,12). Liftover tools are easy to use, therefore users generally assume that the software would generate the correct answer and are not aware of any potential limitations. In fact, previous research had reported discordance and suggested caution when converting genomic variants between assembly versions (13).

Several different tools have now been developed to perform liftover genomic conversion, summarized in Table 1. The software associated with the different liftover tools can be classified into two approaches: (i) Integrity-preserved, a strategy to preserve the length of the overall segment which is the continuity of blocks to ensure the number of bases after lifted with an acceptance threshold and (ii) Non integrity-preserved, that breaks some segment into smaller intervals and convert the intervals independently when there are discordance between the assemblies (14). Although most of the tools were built on top of *UCSC liftOver* with some modifications, it is unclear whether their outputs are identical nor the limitations of each tool. In this study, we benchmark these six commonly used liftover tools; *flo* (15), *CrossMap* (16), *rtracklayer::liftOver* (https://www.bioconductor.org/help/workflows/liftOver), *UCSC liftOver* (17), *segment_liftover* (*segment*) (14) and *NCBI Remap* between human reference genomes hg19 and hg38. We compare the differences between liftover and alignment outputs in terms of coordinates, coverage and DNA methylation characteristics on over 43 WGBS samples. Moreover, the impact of liftover tools on the number and width of segments were evaluated using 366 ChIP-Seq of histone modifications and transcription factors. We also compare the advantages and disadvantages between the two types of algorithms, with and without integrity-preservation and propose a three-step guideline with implementation to enhance genome conversion based on the rationale behind the disruption of liftover.

**Table 1.** List of liftover tools

Name	Chain file UCSC (hg19/hg38)	Tool built on top of the UCSC liftOver	Webtools	Standalone	Input	Integrity preserve	Limit number of rows	Target assembly continuous
***UCSCliftOver***	Yes		https://genome.ucsc.edu/cgi-bin/hgLiftOver	C	BED, GFF/GTF	No	Unlimited	Not continuous
***rtracklayer*::*liftOver***	Yes	Yes		R	BED	No	Unlimited	Not continuous
***CrossMap***	Yes		http://asia.ensembl.org/Homo_sapiens/Tools/AssemblyConverter?db=core	Python	SAM/BAM, Wiggle/BigWig, BED, GFF/GTF, VCF	No	Unlimited	Not continuous
***NCBIRemap***	No, use NCBI remap alignment		https://www.ncbi.nlm.nih.gov/genome/tools/remap	Perl (API)	BED, GFF/GTF, VCF	Yes	250.000 rows	Continuous
***flo***	Yes/Build its own alignment	Yes		Ruby	GFF	No	Unlimited	Not continuous
***segment_liftover***	Yes	Yes		Python	BED, TSV	Yes	Unlimited	Continuous

## MATERIALS AND METHODS

### WGBS data

A total of 43 WGBS samples were downloaded. In details, 41 samples mapped on hg19 and hg38 were downloaded from The Canadian Epigenetics, Environment and Health Research Consortium (CEEHRC) (Supplementary Table S2) (http://www.epigenomes.ca/data-release/hg19/, http://www.epigenomes.ca/data-release/hg38/). LNCaP and PrEC samples were obtained from Pidsley *et al.* (18).

### ChIP-Seq data

A total of 366 ChIP-Seq samples were collected, of which 348 samples were obtained from The Canadian Epigenetics, Environment and Health Research Consortium (CEEHRC) (http://www.epigenomes.ca/data-release/hg19/, http://www.epigenomes.ca/data-release/hg38/). (Supplementary Table S3) and 18 samples of TF originally from Technological Advances for Genomics and Clinics (TAGC) (19).

### Annotation data

All CpG sites (∼28 million CpGs) of hg19 and hg38 were obtained from R package *RnBeads.hg19* (20,21) and *RnBeads.hg38* (21,22), respectively.

Comprehensive gene annotation of human genome on hg19 (ftp://ftp.ebi.ac.uk/pub/databases/gencode/Gencode_human/release_33/GRCh37_mapping/gencode.v33lift37.annotation.gtf.gz) and hg38 (ftp://ftp.ebi.ac.uk/pub/databases/gencode/Gencode_human/release_33/gencode.v33.annotation.gtf.gz) were obtained from GENCODE.

Annotation of common SNPs allele frequencies and and repeat elements were obtained from UCSC for hg19 (http://hgdownload.cse.ucsc.edu/goldenpath/hg19/database/snp150Common.txt.gz, http://hgdownload.cse.ucsc.edu/goldenpath/hg19/database/rmsk.txt.gz) and hg38 (http://hgdownload.cse.ucsc.edu/goldenpath/hg38/database/snp150Common.txt.gz, http://hgdownload.cse.ucsc.edu/goldenpath/hg38/database/rmsk.txt.gz).

Blacklist was downloaded from ENCODE Project Consortium (http://mitra.stanford.edu/kundaje/akundaje/release/blacklists/hg38-human/hg38.blacklist.bed.gz, https://www.encodeproject.org/files/ENCFF001TDO/@@download/ENCFF001TDO.bed.gz).

CpG features (islands, shelves and shores) (23), genic features (3UTRs, 5UTRs, cds, exonintronboundaries, exons, firstexons, intergenic, intronexonboundaries, introns and promoters) (24) were obtained from R package *annotatr* (25).

### Chain files

Chain files to convert from hg19 to hg38, and from hg38 to hg19 were obtained from UCSC (http://hgdownload.soe.ucsc.edu/goldenPath/hg19/liftOver/hg19ToHg38.over.chain.gz, http://hgdownload.soe.ucsc.edu/goldenPath/hg38/liftOver/hg38ToHg19.over.chain.gz)

In-house custom script was used to convert chain file to BED format of 4 categories (Ungapped, gapped-in-hg19, gapped-in-hg38 and gapped-in-both).

### Liftover tools

A total of six liftover tools were used: *crossmap* (version 0.3.0) (http://crossmap.sourceforge.net/) (16), *flo* (https://github.com/wurmlab/flo) (15), *rtracklayer::liftOver* (version 1.8.0) (https://www.bioconductor.org/help/workflows/liftOver/), *UCSC liftOver* (http://hgdownload.soe.ucsc.edu/admin/exe/linux.x86_64/liftOver) (17), *segment_liftover* (https://github.com/baudisgroup/segment-liftover) (14) and *remap* (https://www.ncbi.nlm.nih.gov/genome/tools/remap).

All tools were run on a machine with Intel Xeon CPU E5 2.20 GHz with 32 cores, 32 GB of RAM running Ubuntu 16.04. Using *memory_profiler* (https://github.com/pythonprofilers/memory_profiler), the maximum memory requirements for WGBS conversion of all the tools were estimated as 18 MB.

### WGBS alignment analysis

Bisulfite reads of LNCaP and PrEC were preprocessed, aligned and DNA methylation calling by *Meth10X* (https://github.com/luuloi/Meth10X) (3).

### Differential methylation analysis

Differential methylation analysis was performed using two methods: delta value and *MethPipe*. Delta value was obtained from subtracting DNA methylation value of each CpGs of alignment output from liftover output, with delta ≥ 0.2 was defined as significant difference. *MethPipe* was used to call DMRs/DMCs.

### Shrink or extend interval

Segment before (B) and after (A) liftover is defined as equal width when they satisfy the following criteria $\frac{1}{n} < \frac{{{\rm width}( {\rm B} )}}{{{\rm width}({\rm A} )}} < n$. If $\frac{{{\rm width( B} )}}{{{\rm width( A} )}} >n$, this segment is defined as shrink. If $\frac{{{\rm width( B} )}}{{{\rm width( A} )}} < \frac{1}{n}$, this segment is defined as extend, where *n* is chosen as 2 in our analysis.

### Enrichment analysis

There are four steps to conduct enrichment analysis. First, finding the overlapping between interested CpGs/intervals and 20 genomic annotations (Islands, shelves, shores, 3UTRs, 5UTRs, cds, exonintronboundaries, exons, firstexons, intergenic, intronexonboundaries, introns, promoters, repeat, snp150Common, blacklist, ungapped, gapped-in-hg19, gapped-in-hg38 and gapped-in-both) with *bedtools* and count frequency. Second, randomizing the interested CpGs/intervals on ∼28 million CpGs/genome wide to generate the new random coordinates. Third, intersecting these random coordinates with 20 genomic annotations and count frequency. These counts are put into the two by two contingency table. At last, one-sided Fisher’ exact test is applied to the table to check the enrichment of the interested CpGs/intervals on these 20 genomic features.

### Kappa statistics (agreement test)

Kappa statistics is used to investigate the agreement between two outputs (liftover versus alignment). With 43 methylomes, we categorized coverage values into five bins of (0–6), (6–12), (12–18), (18–24) and (24–32); and classified DNA methylation values into five bins of (0.0–0.2), (0.2–0.4), (0.4–0.6), (0.6–0.8) and (0.8–1.0). These bins were plotted as a heatmap using gplots (26). Finally, Kappa statistics was calculated to measure the agreement between two outputs using function kappa2 (unweighted) in IRR package. Kappa values ranging from −1 to 1 were interpreted as follows: values <0.0 indicating no agreement, +0.0 to 0.2 indicating slight agreement, +0.21–0.40 indicating fair agreement, +0.41–0.60 indicating moderate agreement, +0.61–0.80 indicating substantial agreement and +0.81–1.0 indicating perfect agreement. The same procedure was applied to 18 transcription factor ChIPseq to evaluate the agreement between alignment and liftover of transcription factor binding sites.

### Jaccard statistics

We use Jaccard statistics which is implemented in *bedtools* to measure the similarity of the two sets based on the intersections between alignment and liftover of epigenome/ChIPseq data.

$$\begin{equation*}{\rm Jaccard }\left( {A,B} \right) = \frac{{{\rm length}\left( {A\ {\rm Intersects}\ B} \right)}}{{{\rm Sum\ of\ length}\left( {A + B} \right) - {\rm length}\left( {A\ {\rm {\rm Intersects}}\ B} \right)}} \end{equation*}$$

*A* and *B*: two sets of genomic intervals.

### Statistic tests

Two-sided *t*-test, one-sided Fisher's exact test, Pearson correlation and unweighted Kappa statistics are computed using R version 3.5.0.

MDS plot was generated by ggplot2 in R. Unsupervised hierarchical clustering was illustrated by heatmap using R package ComplexHeatmap (https://bioconductor.org/packages/release/bioc/html/ComplexHeatmap.html) (27).

All the other plots are generated using package ggplot2, graphics (smoothScatter) in R version 3.5.0.

### Availability of data and materials

All track data of gapped-in-hg19, gapped-in-both, gapped-in-hg38, blacklist, not CG and all the scripts used in this study are available in github (https://github.com/phuoc362/Lifted).

## RESULTS

### Liftover tool summary

**Figure 1.**
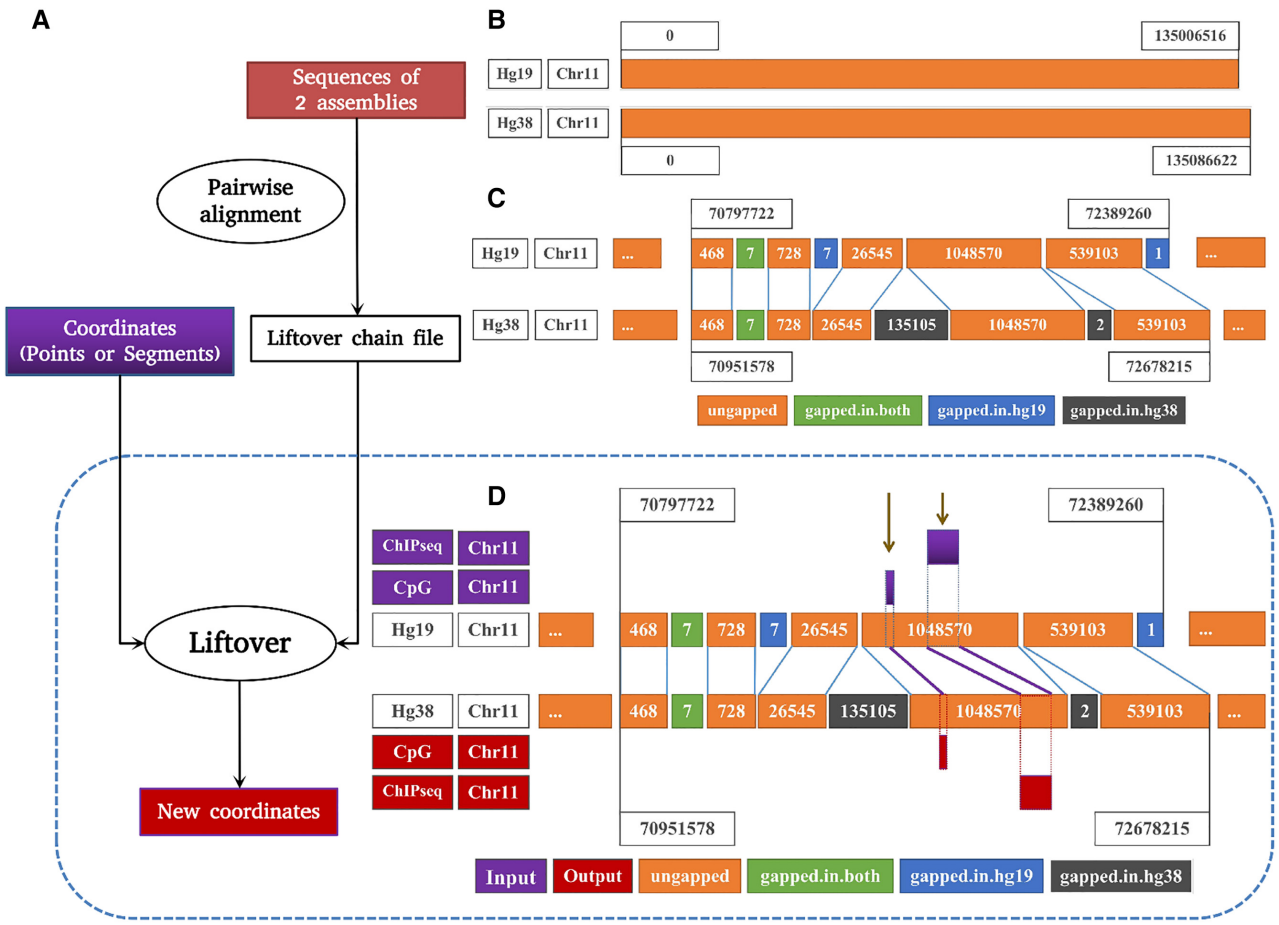
Summary of liftover conversion. (**A**) Detailed steps of the liftover process. (**B**) An example of discordance between two reference assemblies hg19 and hg38. (**C**) Ungapped, gapped-in-hg19, gapped-in-both, gapped-in-hg38 regions and the principle of conversion between reference genomes. (**D**) Results of liftover CpGs and ChIP-Seq data on the ungapped region.

Several different tools have now been developed to perform liftover genome conversion, summarized in Table 1. Among the six commonly used tools, only *flo* (15) can perform the entire conversion pipeline, as presented in Figure 1A. *Flo* allows users to liftover coordinates from scratch by generating its own alignment format using *BLAT* (28), whereas the other tools require a chain file. However, *flo* is relatively time consuming and requires intensive computational processes, but it is useful when genomic alignments are not available. The algorithms of all 6 tools are consistent for point inputs as they do not need to preserve the length of the point. Whereas for the segment inputs, the integrity of the length of the sequence and continuity of block has to be preserved. *CrossMap* (16), *flo*, *rtracklayer::liftOver* and *UCSC liftOver* (17) follow the same strategy of breaking the segments into smaller intervals when they cross discordant blocks in the underlying alignments. They then map each interval to the target assembly and merge the new coordinates until a gap is met. If the gap width is less than the input segment width, the process will be continued. As a result, an input segment could be sheared into multiple smaller segments after conversion. In contrast, *segment_liftover* (*segment*) (14) and *NCBI Remap* are integrity-preserving, as they try to maintain integrity of the interval and map its entire span to the target assembly using specific criteria. *Segment* is a tool built on top of the *UCSC liftover* with some criteria to adjust before giving final results.

### Principle of liftover alignment format

Before liftover conversion can be conducted, pairwise alignment needs to be performed between two genome assemblies—source assembly and intended assembly in FASTA format as inputs (Figure 1A). Differences between the two assemblies are recorded, for example chromosome 11 as shown in Figure 1B, and the output from this step is used to produce the alignment format, such as UCSC chain file (through *BLAT* tool (28)) or NCBI assembly–assembly alignments (through *BLAST* tool (29)). Both are available for model organisms, such as genome assemblies for human, mouse, zebrafish and drosophila. The alignment format between genome builds contain the coordinates of gapped and ungapped segments, without the DNA sequence. Liftover tools then use the alignment format to convert coordinates, either points or segments (purple colored in Figure 1A and D), between the two assemblies and generate new coordinates (red colored in Figure 1A and D).

**Figure 2.**
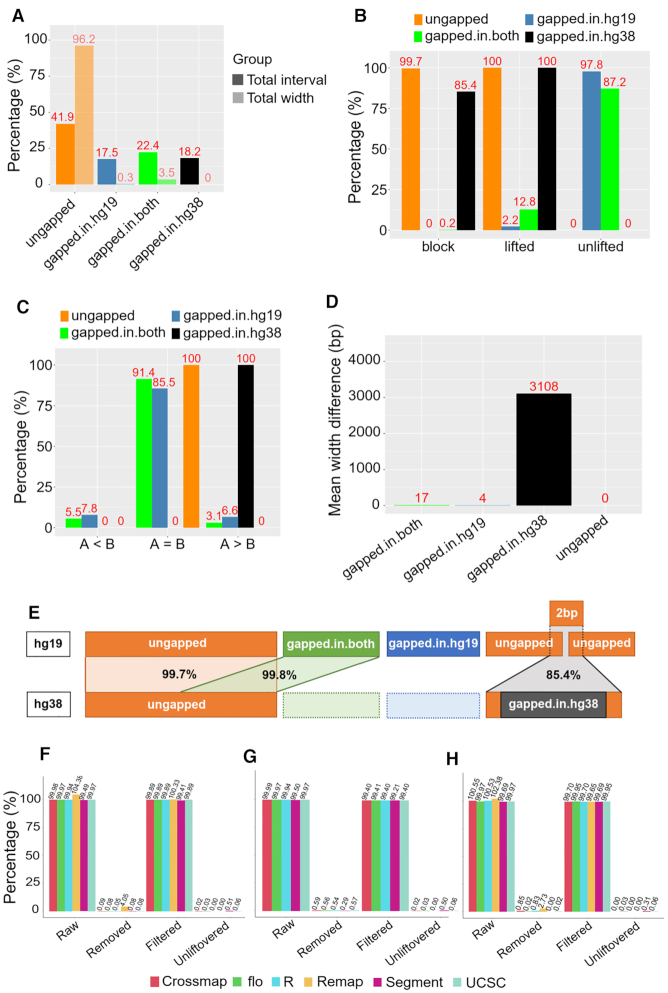
Benchmarking six liftover tools from hg19 to hg38. (**A**) Percentage of the number of intervals and length of ungapped, gapped-in-hg19, gapped-in-both and gapped-in-hg38 on the hg19 assembly. (**B**) Percentage of lifted, unlifted and lifted to the corresponding block by regions. (**C**) Comparing the length of intervals before and after conversion by regions. (**D**) Mean of width difference by regions. (**E**) Summary of the underlying principle of liftover for each region. (**F**) Liftover 250 000 CpGs using six tools. (**G**) Liftover all CpGs using five tools (exclude *NCBI remap*). (**H**) Liftover a ChIP-Seq sample using six tools.

In order to benchmark the six different liftover tools, we first analyzed the UCSC chain file used for the genome alignment of hg19 to hg38 to understand the underlying principle of genome conversion. The chain file segmented the two genome assemblies into multiple blocks with the direction of strand (for simplification this is not presented on Figure 1). The blocks can be grouped into four categories based on the alignment that allow gaps in both sequences concurrently (https://genome.ucsc.edu/goldenPath/help/chain.html). Ungapped (orange colored in Figure 1C), the most frequent region that accounted for 96.2% of the total length of hg19, contains all the well-matched blocks between the two assemblies. A minor amount of contigs on hg19 (17.5% of the number of blocks comprising only 0.3% the length of the genome) cannot be mapped to hg38 and these form regions we call gapped-in-hg19 (blue colored in Figure 1C). Conversely, the hg38 build contains more assembled sequences that do not exist in hg19, which we call gapped-in-hg38 (black colored in Figure 1C). Finally, the regions where corresponding coordinates of hg19 and hg38 both contain gaps we defined as gapped-in-both (green colored in Figure 1C) (3.5% of the length of genome) (Figure 2A).

*UCSC liftOver* was used to convert each region, to investigate their conversion characteristics. However, since the gapped-in-hg38 does not exist in the hg19, we used simulation to generate the corresponding intervals on hg19 that stretched across gapped-in-hg38. First, to detect the coordinates of gapped-in-hg38 on hg19, all the blocks on hg19 were divided into two files, one contained the gapped, the other contained all the ungapped sequences. Normally two ungapped blocks are separated by a gapped-in-hg19 (their coordinates should be interrupted). Therefore, in the ungapped file, if there are two blocks situated next to each other but their coordinates are continuously connected, that is where the gapped-in-hg38 will insert later. All the detected coordinates were added 1 bp to both directions to make blocks of 2 bp that use the gapped-in-hg38 coordinates as the midpoint.

We showed that 100.0% ungapped blocks were successfully converted (Figure 2B), of which 99.7% were lifted to the correct corresponding region on hg38. The gapped blocks were mostly unlifted. A small percentage of gapped-in-both (12.8%) and gapped-in-hg19 (2.2%) were lifted, but were potentially mapped to inappropriate regions as these coordinates are not in hg38. Only 0.2% of the gapped-in-both, and 0.0% of the gapped-in-hg19 were lifted to the corresponding region on hg38. All of the gapped-in-hg38 (100.0%) lifted, but this is due to the simulation technique where these intervals are actually the 2 bp ungapped blocks in hg19 that crossed the gapped-in-hg38. We next compared the width of blocks before (B) and after (A) liftover (Figure 2C) to understand more fully the conversion attributes of this region. Ungapped, gapped-in-hg19 and gapped-in-both still maintain their width after conversion, as notably all of the ungapped blocks (100.0%) had their size preserved (A = B). Interestingly, all of the gapped-in-hg38 blocks (100.0%) showed a rise in size (A > B), corresponding to an average increase of 3108 bp (Figure 2D). Considering all of these simulated intervals have an original width of only 2 bp, our data show that the blocks crossing gapped-in-hg38 regions are always converted into corrupted segments with much larger insert sizes. Figure 2E summarizes the mapping efficiencies and caveats associated with the liftover chain file we found from our analyses above.

### Similar performance was observed among liftover tools

To benchmark the performance across six widely used liftover tools (*USCS liftOver, rtracklayer::liftOver*, *CrossMap, NCBI Remap, flo* and *segment_liftover*) summarized in Table 1, we first lifted 250 000 random CpGs from hg19 to hg38 to compare the tools. Raw output was defined as successfully converted CpGs directly obtained from the different liftover tools. Any CpG that could not be converted were defined as unliftovered. Inappropriate data from raw was removed, including (i) duplication which was defined as any CpG on hg19 that was converted to more than one CpG on hg38; (ii) CpG aligned on alternative chromosomes; and (iii) not CG, that is any CpG on hg19 that no longer occurred after conversion on the hg38 build. We obtained filtered output from subtracting removed data from raw output. The proportion of raw output compared to input data ranged from 99.49 to 104.38%, with the only figure above 100.00% generated by *NCBI Remap*. After removing inappropriate data, the average filtered data accounted for 99.89% of the input number of CpGs, indicating adequate conversion accuracy among all tools (Figure 2F). To confirm the liftover accuracy, we converted the same 250 000 CpGs coordinates from hg38 back to hg19, and also found consistent results among six tools (Supplementary Figure S1a).

Although *NCBI Remap* is an integrity-preserving conversion tool, it limits 250 000 rows to being lifted-over at once, which only accounts for 0.89% of ∼28 million CpGs in total. For this reason, we excluded *NCBI remap* from our comparison of the conversion of all CpGs in hg19 and hg38, using the other five tools in Table 1. Results for CpG sites across the whole genome again showed that the tools gave similar output, confirming similar functioning among the tools (Figure 2G). Results of converting hg38 to hg19 were also consistent (Supplementary Figure S1b).

Since the algorithms of some of the tools theoretically perform better on segments, we used the same process to assess whether there are any differences between converting points and intervals, regarding interval conversion. We used a representative ChIP-Seq sample (H3K4me1_CEMT0009) that contains the highest number of segments among 366 ChIP-Seq samples of 147 398 and as this was less than 250 000, we were able to compare conversion with all six tools. The results were consistent with those of point data, with 99.69–102.38% segments converted and 99.69–99.95% segments after removing inappropriate data (i.e. duplication and alternative chromosome as defined above) regarding hg19 to hg38 (Figure 2H). Results of converting hg38 to hg19 were also identical (Supplementary Figure S1c).

We conclude that conversion using any of the six liftover tools are relatively similar in point (WGBS) and segment (ChIP-Seq) data. We suggest that this is because all of these tools are based on the same UCSC chain file produced from pairwise alignment between hg19 and hg38.

### Liftover mapping correct feature location onto the new assembly

**Figure 3.**
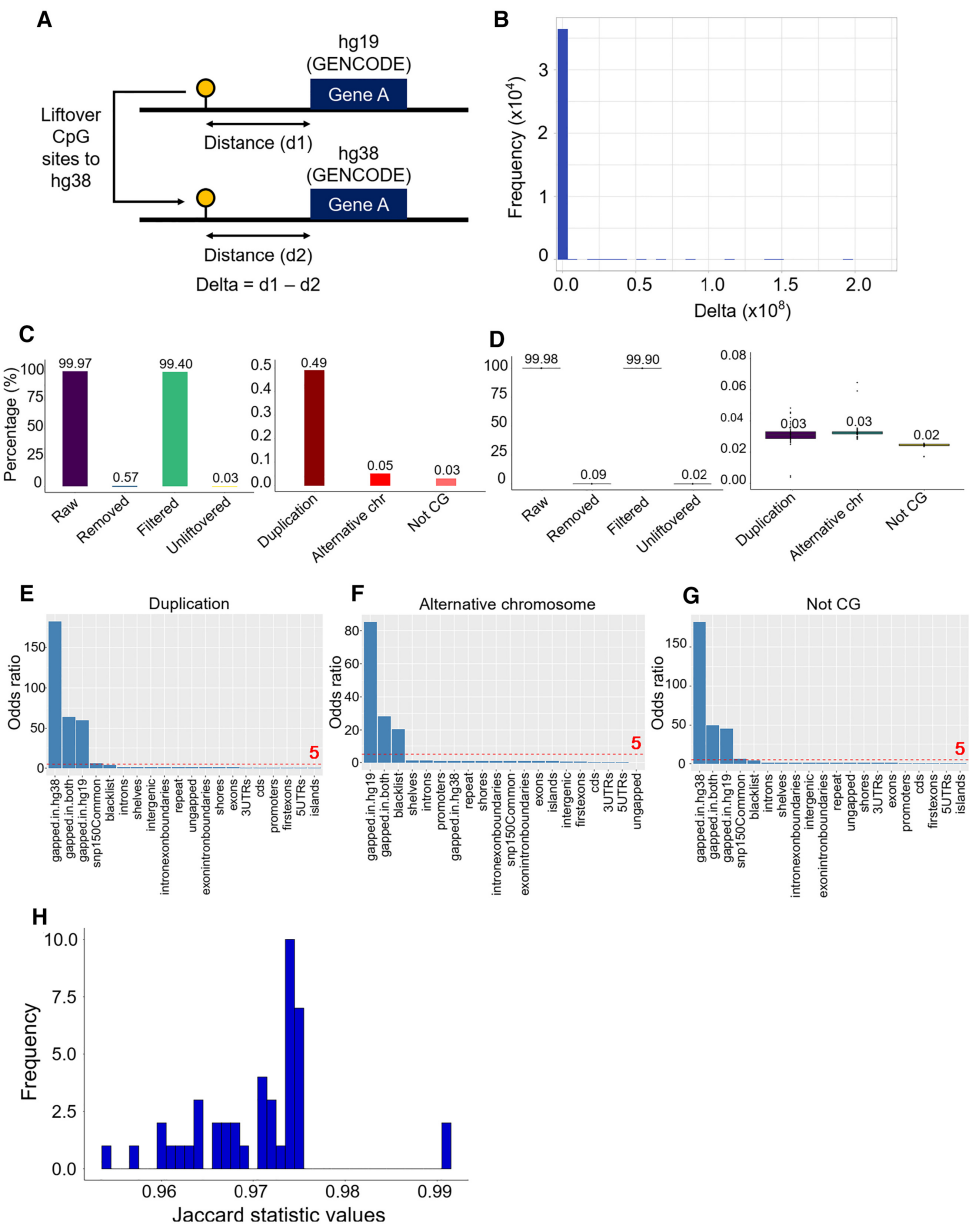
*UCSC liftOver* conversion of WGBS data. (**A**) Steps of analysis to determine if CpG sites are mapped to the correct location. (**B**) Distribution of delta values by subtracting distance between initial CpGs and gene coordinates on hg19 (d1) by distance between lifted CpGs and gene coordinates on hg38 (d2). (**C**) *UCSC liftOver* conversion of the full CpG dataset and details of removed data. (**D**) *UCSC liftOver* conversion of 43 WGBS samples and details of removed data. (**E**) Enrichment analysis of CpG duplication. (**F**) Enrichment analysis of CpG alternative chromosome. (**G**) Enrichment analysis of not CG. (**H**) Distribution of Jaccard statistic values of corresponding CpG coordinates between liftover and alignment methylomes among 43 WGBS samples.

Since liftover tools only process genome positions, we determined whether features are also mapped to correct location. In this analysis, we investigated CpG sites as the feature and whether or not its coordinates are accurately converted by comparing the distance between CpG sites to the closest genes, before and after liftover (Figure 3A). Gene annotation on hg19 and hg38 generated by GENCODE (30) were used as the landmark for this comparison. First, we selected concordant genes that have the same length in both hg19 and hg38 and exported their coordinates on hg19 and hg38, respectively. Using the full CpGs dataset from the reference genome hg19, we detected the gene on hg19 that was closest too (but not covered) by each CpG site using *bedtools* and computed the distance (d1). After removing duplication, a total of 36 498 pairs of unique genes and the CpG site with minimum distance were recorded. We then converted the coordinates of CpGs in these pairs to hg38 using *UCSC liftover*. Distance between each lifted CpG and the corresponding gene pair with hg38 coordinates (d2) was measured. We obtained the delta value by subtracting the distance between the initial CpG and gene coordinates on hg19 (d1) by distance between lifted CpGs and gene coordinates on hg38 (d2). We found that the majority (99.3%) of the delta values was 0, which indicated that the conservation of the relative relationship between CpG sites and their closest genes was preserved (Figure 3B).

### Efficient conversion of epigenome data between liftover and alignment output

To next investigate if the conversion process from hg19 to hg38 can affect the coordinates of point data, *UCSC liftOver* was used to convert the full CpG dataset from the reference genome hg19, which is the maximum number that a WGBS sample can reach. *UCSC liftOver* successfully converted 99.97% of the full CpG dataset. After removing the duplicated data (0.49%) and alternative chromosome (0.05%) and not CG (0.03%), respectively the filtered data was left with 99.40% of all hg19 CpG coordinates (Figure 3C). In principle due to the nature of the DNA methylome, WGBS data from individual clinical samples will not retain all CpG sites after bisulphite conversion, as in non-bisulphite reference genome. To test the performance of genome conversion on DNA methylome data, we converted the coordinates from 43 WGBS clinical samples using *UCSC liftOver*. The results were similar to converting the full CpGs dataset with a median of 99.98% CpGs successfully converted. The liftover was remarkably accurate since only 0.09% had to be removed, as the proportion of duplication, alternative chromosome and not CG were relatively equal, ranging from 0.02 to 0.03% (Figure 3D).

To explore the location of the 0.09% inappropriate data, we used one-sided Fisher's exact test a set of 20 genomic features (Figure 3E–G). Odds ratio (OR) of 5.0 and *P*-value < 0.001 was used as the significance threshold. We found that duplication was enriched (*P*< 0.001) in three gapped regions (OR = 59.441–181.946) (Figure 3E). The same pattern was observed in not CG (OR = 45.161–181.946 for three gapped regions) (Figure 3G) and alternative chromosome was significantly enriched in gapped-in-hg-19 (OR = 85.333), gapped-in-both (OR = 28.088) and blacklist (OR = 20.277) (Figure 3F). Enrichment in the blacklist was probably due to artifacts of alignment that have yet to be filtered. Supplementary Table S4 summarizes the rest of genomic features with an OR of <5.0.

Finally, data aligned to hg38 were downloaded to evaluate the similarity of CpGs coordinates between liftover and alignment outputs. Jaccard statistics was computed among the 43 WGBS datasets using bedtools (31) (see ‘Materials and Methods’ section for details). With a minimum overlap requirement set to 1 bp, Jaccard statistic values ranging from 0.96 to 1.00, suggested there were only minimal differences between the two approaches (Figure 3H).

### Coverage and DNA methylation profiles are essentially preserved between liftover and alignment approaches

As the outputs from genome conversion were found to be similar between liftover and alignment, we next investigated if the liftover process can have an effect on annotation data, such as coverage and DNA methylation. To determine how the accuracy of coverage and DNA methylation calling compares between the liftover approach and re-alignment approach, we assessed the concordance of coverage and DNA methylation levels between liftover and alignment outputs based on two measures, correlation (using Pearson correlation coefficient) and agreement (using kappa statistic (32)).

**Figure 4.**
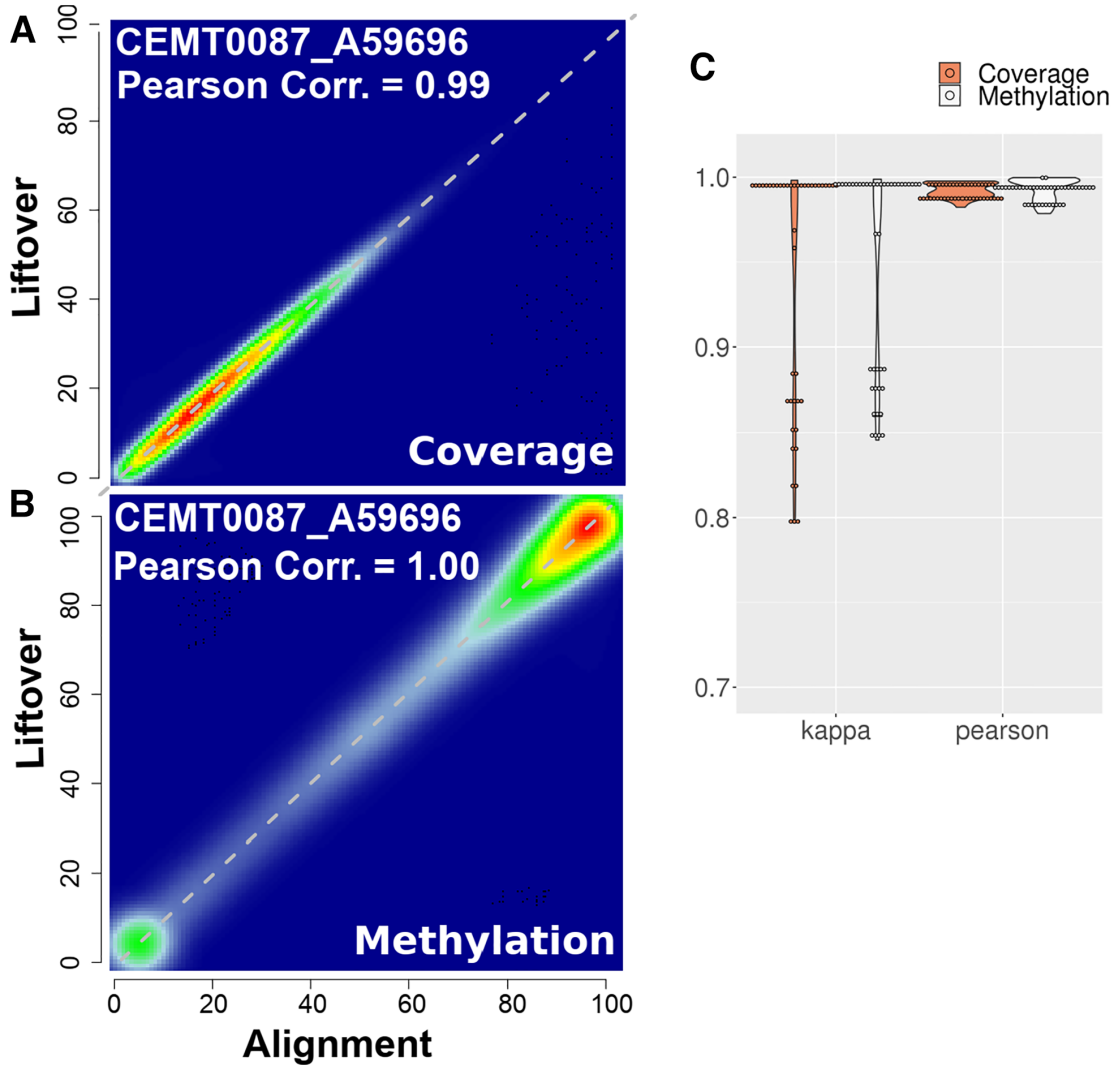
Liftover preserves coverage and DNA methylation of epigenome. (**A**) Scatter plot of coverage values between liftover and alignment methylome of sample CEMT0087_A59696. (**B**) Scatter plot of DNA methylation values between liftover and alignment methylome of sample CEMT0087_A59696. (**C**) Violin plot of Pearson correlation coefficients and kappa statistics between liftover and alignment methylomes across 43 samples.

Regarding coverage, the distribution of liftover and alignment outputs were similar (Supplementary Figure S2a). For each sample, we determined the relationship of CpGs coverage on a scatter plot, and calculated the Pearson correlation coefficients. For instance, on sample CEMT0087_A59696, there was a high density of coverage points around the diagonal, *r* = 0.99, indicating an excellent correlation (Figure 4A). We also categorized coverage values into five equally sized bins, and found kappa = 0.99, supporting the strong agreement between the liftover and alignment outputs (Supplementary Figure S2b). In terms of DNA methylation, the same method was used for each pair of liftover and alignment outputs. We obtained similar highly correlated results on sample CEMT0087_A59696 (Figure 4B and Supplementary Figure S2d) between the two approaches and the distribution of DNA methylation values looked similar (Supplementary Figure S2c). The same correlation and agreement analysis was applied on all 43 WGBS samples. Figure 4C shows that there is was strong correlation and agreement regarding coverage and DNA methylation among all 43 samples. Interestingly we found that the depth of coverage and the DNA methylation value are independent of the kappa statistics (Supplementary Figure S2e and f).

**Figure 5.**
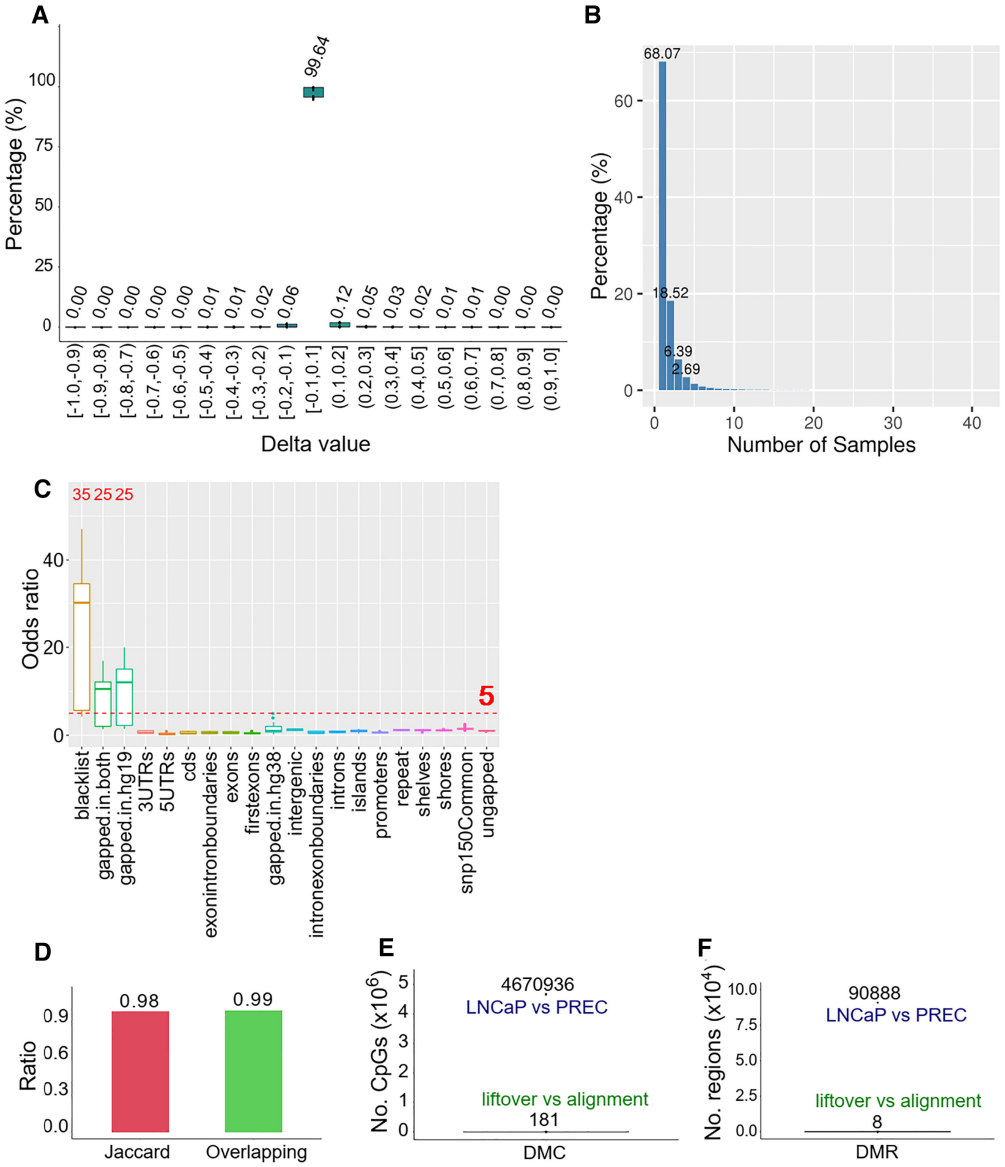
Liftover preserves DMRs. (**A**) Distribution of delta values of CpGs among 43 WGBS samples. (**B**) The percentage of overlapping CpGs with delta ≥ 0.20 among 43 samples. (**C**) Enrichment analysis of CpGs with delta ≥ 0.20 across 43 samples. (**D**) Jaccard statistic and overlapping proportion between DMR1 (liftover-LNCaP versus liftover-PrEC) and DMR2 (alignment-LNCaP versus alignment-PrEC) in hg38. (**E**) DMC sites between a pair of liftover and alignment output in 43 samples called by *MethPipe*, in comparison with DMCs between LNCaP and PrEC. (**F**) DMRs between a pair of liftover and alignment output in 43 samples called by *MethPipe*, in comparison with DMRs of alignment-LNCaP versus alignment-PrEC in hg38.

We next asked if the minor differences due to conversion are potentially called as random error of differentially methylated regions (DMRs) or differentially methylated CpGs (DMCs). We performed differential methylation analysis using two methods, delta value and the *MethPipe* tool (8). In each sample, we first obtained delta values for each CpG by subtracting the DNA methylation level of alignment from the liftover output. For example, on sample CEMT0087_A59696, we found delta values mostly concentrated around 0.0 (Supplementary Figure S3a). Performing this process across all 43 WGBS data, we found that 99.64% of delta values were distributed at 0.0 as presented in Figure 5A, indicating the majority of the methylation value differences between liftover and alignment outputs were not significant. To clarify whether the significant differential methylation (delta ≥ 0.2) occurred at the same CpG site among 43 samples, we counted how many times the same coordinates of CpGs with delta ≥ 0.2 was observed across the 43 samples between liftover and alignment outputs. The percentage of occurrences is presented in Figure 5B and frequency values in Supplementary Figure S3c. The data indicates that 68.07% of these CpGs are unique. The percentage considerably dropped to 18.52% if the CpGs occurred concurrently in two samples, 6.39% in three samples and continuously decreased to approximately zero. Therefore the CpGs with delta ≥ 0.2 are only unique to each sample. In short, the DNA methylation call of each CpG site does not significantly change after liftover.

We next investigated whether CpGs with significant methylation differences are enriched in specific genomic features. Using CEMT0062_A59692 as an example, one-sided Fisher's exact test was used to test for enrichment with the same method and significant threshold, (see ‘Materials and Methods’ section). In this sample, blacklist (OR = 33.694), gapped-in-hg19 (OR = 19.741) and gapped-in-both (OR = 16.895) were found as the significant (*P*< 0.001) enriched features, while the OR of all other features were <5.0 (Supplementary Figure S3b). We continuously applied enrichment analysis on all WGBS samples (Figure 5C). Substantial enrichment was confirmed in the blacklist (median OR = 30.119 with 35 significant samples), gapped-in-hg19 (median OR = 12.036 with 25 significant samples) and gapped-in-both (median OR = 10.517 with 25 significant samples), the rest of features were not significant. Again, enrichment in the blacklist is due to unfiltered alignment data rather than the liftover process. The main causes of methylation differences are gapped-in-hg19 and gapped-in-both, since they generally shift to inappropriate positions after being lifted. Details of median OR for all features can be found at Supplementary Table S5.

### Differential methylation observed between liftover and alignment from the same WGBS samples show a high degree of overlap

We next asked if the liftover process impacts differential DNA methylation calling between two different DNA methylomes by comparing WGBS data from normal prostate cells (PrEC) and prostate cancer cell line (LNCaP). We used *MethPipe* to detect DMRs using the *UCSC liftOver* version from hg19 to hg38 (DMR1) from LNCaP and PrEC, and we also called DMRs with the hg38 WGBS alignment outputs from LNCaP and PrEC (DMR2). The DMR2 alignment outputs was confirmed in MethylationEPIC BeadChip microarray (18). The overlap between DMR1 and DMR2 was measured and divided by the average of DMR1 and DMR2 to determine the concordance between the two outputs. The data showed a high (99.13%) overlap between DMR1 and DMR2. Jaccard statistic was calculated using *bedtools* to measure the similarity of DMRs distribution. Jaccard statistic value was found 0.98 indicating similarity between the DMR calling from the liftover and alignment outputs (Figure 5D).

**Figure 6.**
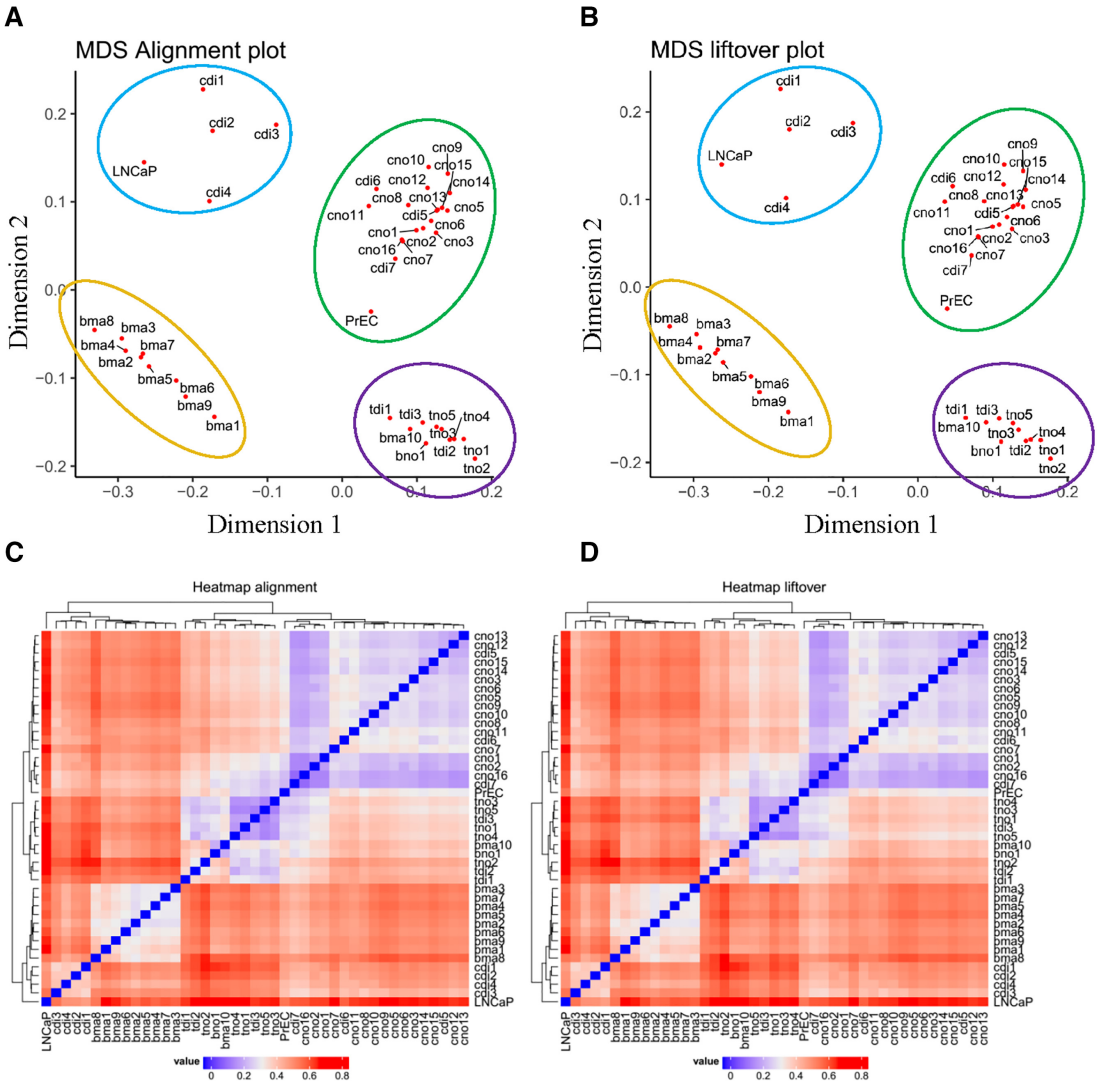
Liftover maintains DNA methylation signature of cell type specificity. (**A**) MDS of 43 alignment methylomes. (**B**) MDS of 43 liftover methylomes. (**C**) Unsupervised hierarchical clustering of 43 alignment methylomes. (**D**) Unsupervised hierarchical clustering of 43 liftover methylomes.

Finally, we used *MethPipe* to perform DNA methylation analysis for each pair of liftover and alignment across the 43 samples and the results were compared to a control value, that is the number of DMRs/DMCs obtained between LNCaP and PrEC WGBS data. On average, there were only 8 (4–20) DMRs and 181 (99–403) DMCs detected, in comparison with 90 888 DMRs and 4 670 935 DMCs detected from LNCaP and PrEC, respectively (Figure 5E and F). We were interested to determine in these differences could affect the outcome of clustering similar samples using DNA methylation values. Among 43 samples, methylation values of all CpGs were combined to perform multi-dimensional scaling (MDS) and unsupervised hierarchical clustering. The MDS plots showed similarity between liftover and alignment outputs. The samples were clustered on four quadrants according to sample origin (Figure 6A and B). The same results were obtained from hierarchical clustering (Figure 6C and D). Therefore, in general the liftover procedure did not substantially change the clustering profile from the alignment profile of DNA methylation.

### *Segment* outperformed *UCSC* in interval conversion in regards to the width of ChIP-Seq segments

While all tools followed the same strategy to liftover point data, the algorithms to convert segment data, such as ChIP-seq data, can be classified to two groups. *UCSC liftOver, rtracklayer::liftOver, CrossMap*, *flo* tend to break the segment into smaller pieces and merge them after conversion, while *NCBI Remap* and *segment_liftover* use an integrity-preserving strategy. As there is no reference data for ChIP-Seq, we converted 366 ChIP-Seq samples from hg19 to hg38 following a similar process as for processing the WGBS, using two representative tools *UCSC liftOver* and *segment* to compare the number of converted interval between the two types of algorithms.

**Figure 7.**
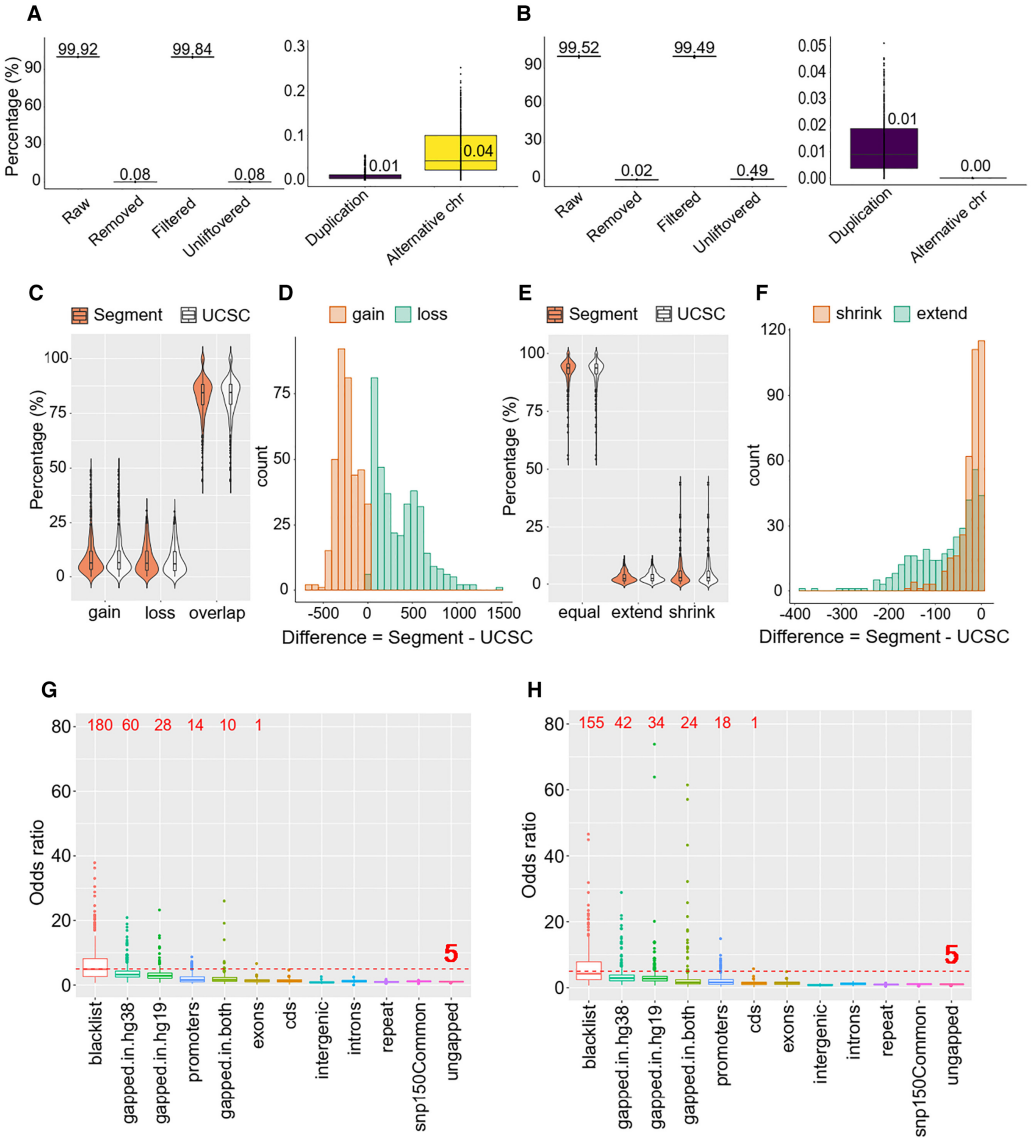
*UCSC liftOver* and *segment_liftover* conversion of 366 ChIP-Seq samples. (**A**) *UCSC liftOver* conversion and details of removed data. (**B**) *segment_liftover* conversion and details of removed data. (**C**) Violin plot showing the percentage of gain, loss and overlapping intervals. (**D**) Histogram showing differences of number of gain and loss intervals between *segment_liftover* and *UCSC liftOver*. (**E**) Violin plot showing the percentage of equal, extend and shrink intervals. (**F**) Histogram showing differences of number of intervals with extend and shrink in length between *segment_liftover* and *UCSC liftOver*. (**G**) Enrichment analysis of loss and gain intervals in *UCSC liftOver* liftover versus alignment outputs. (**H**) Enrichment analysis of loss and gain intervals in *segment_liftover* liftover versus alignment outputs.

In terms of *UCSC liftOver*, there were 99.92 (99.88–99.94)% successfully converted segments, of which only 0.08 (0.05–0.13)% had to be removed. Removed data were mostly due to mapping on alternative chromosomes, with a median of 0.04 (0.02–0.10)%, as presented in Figure 7A. Considering *segment_liftover*, the number of raw intervals was slightly lower with 99.51 (99.31–99.62)%, corresponding to 0.49 (0.38–0.67)% unconvertible. However, there was only 0.02 (0.01–0.05)% inappropriate data, of which no interval was mapped to an alternative chromosome, the only reason for removing was duplication (Figure 7B). This is because of the tool algorithm; *segment_liftover* will only map an interval when its width ratio, before and after liftover, ranges from 0.5 to 2.0 and the new coordinates must be located on the same chromosome. Any segments that do not satisfy these criteria are added to unliftover group. We found that the proportion of filtered intervals using *UCSC liftOver* (99.84 (99.72–99.88)%) was higher than using *segment_liftover* (99.49 (99.25–99.60)%), however we cannot conclude which tool is more accurate without further investigating other features of these filtered intervals.

To therefore comprehensively investigate the impact of conversion on interval data, we evaluated the differences in the number and width of intervals between liftover and alignment outputs. Alignment outputs were treated as the standard. We defined changes in the number of intervals as follows: (i) loss: interval that exists in alignment output but does not exist in liftover output; (ii) overlapping: interval that fully exists in both alignment output and liftover output; (iii) gain: extra interval that newly appears in liftover output but does not exist in alignment output. Each interval, either in liftover or alignment output, was classified into one of these categories. We calculated the percentage of the three categories for each sample. Among 366 ChIP-Seq samples, the overlapping group accounted for the highest percentage, with similar median values between the two tools (84.39% for *segment_liftover* and 84.52% for *UCSC liftOver* results, respectively) (Figure 7C). The difference in the loss and gain groups between *UCSC liftOver* and *segment_liftover* was compared by subtracting *UCSC liftOver* results from *segment_liftover* results (Figure 7D). *Segment_liftover* significantly generated less gain intervals than *UCSC liftOver* (95.90% of difference values ≤0), while the number of its loss intervals were considerably greater (100.00% of difference values ≥0).

Among the overlapping segments, we computed the ratio of width of the segments between liftover output and alignment output to identify the variation of width. Changes were defined as: (i) shrink: the ratio of liftover segment to alignment segment <0.5; (ii) equal: the ratio between the two segments ranging from 0.5 to 2.0; (iii) extend: the ratio of liftover segment to alignment segment >2.0 (14). Details can be found in the ‘Materials and Methods’ section. The median percentage of the equal group was high in both tools (93.73% for *segment_liftover* and 93.70% for *UCSC liftOver*) (Figure 7E). We then subtracted *UCSC liftOver* outputs from *segment_liftover* outputs to measure differences of width of intervals between the two tools. Both the number of extend and shrink intervals from *segment_liftover* are significantly less than *UCSC liftOver* (100% of difference values ≤0) (Figure 7F).

We next performed enrichment analysis using one-sided Fisher's exact test to investigate the specific genomic features that had loss or gain of enrichment. Results showed that enrichment of loss and gain in *segment_liftover* and *UCSC liftOver* followed the same pattern (Figure 7G and H). Although significant enrichment was not observed in any genomic features, we found features with the highest OR were blacklist, gapped-in-hg38 and gapped-in-hg19 for both *UCSC liftOver* and *segment_liftover*. Enrichment in blacklist was caused by unfiltered alignment data rather than the impact of liftover process. Gapped-in-hg38 and gapped-in-hg19 again appeared as the problematic regions, but it is expected since they are the unmatched between the two reference assemblies. Particularly in the problematic gapped-in-hg38 region, while 60 *UCSC liftOver* samples had OR > 5.0, only 42 samples had significant enrichment in this region when *segment_liftover* was used, suggesting that the integrity-preserved algorithm had some impact. Details of median OR for all features can be found at Supplementary Table S6 and Figure S4a and b.

**Figure 8.**
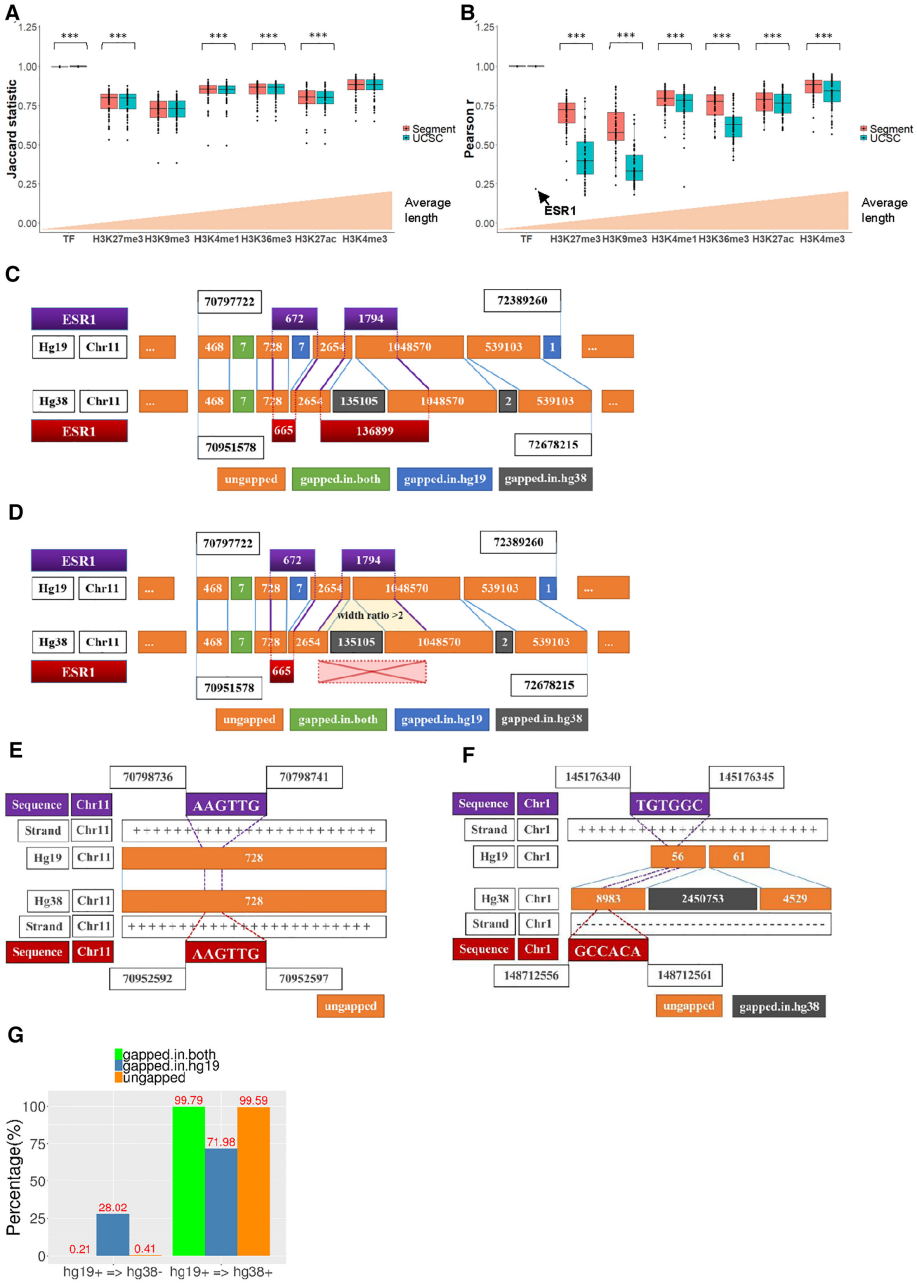
Concordance between liftover and alignment outputs of ChIP-Seq samples using *segment_liftover* and *UCSC liftOver* (***P< 0.001). (**A**) Box plot showing Jaccard statistic of similarity between the two outputs using *segment_liftover* and *UCSC liftOver*. (**B**) Box plot showing Pearson correlation coefficients of intervals length between the two outputs using *segment_liftover* and *UCSC liftOver*. (**C**) Corrupted positions of sample ESR1 were converted by *UCSC liftOver*. (**D**) Corrupted positions of sample ESR1 were filtered by *segment_liftover*. (**E**) Liftover of sequence, a general example. (**F**) Liftover of sequence, a corrupted example. (**G**) Percentage of base pair of contigs unchanged/changed of strand from hg19 to hg38.

We then grouped the ChIP-Seq samples according to the specific type of antibody used to investigate the application of genome conversion on different ChIP-Seq datasets. With a minimum overlap requirement set to 1 bp, we computed Jaccard statistic to measure the similarity of coordinates distribution between liftover output and alignment output for each type of ChIP-Seq data. Figure 8A compares the average Jaccard statistic values between *segment_liftover* and *UCSC liftOver*, which was ordered by the average length of samples in *x*-axis (Supplementary Figure S5a). Significant differences were found between the *segment_liftover* and *UCSC liftOver* conversions in histone modification samples using paired sample *t*-test, however the differential magnitudes are small (0.0076). The data also show that the length of intervals is independent with Jaccard statistic.

Finally, we computed Pearson correlation coefficient to measure the correlation of width of intervals between the two outputs and compared *segment_liftover* to *UCSC liftOver* algorithm. Figure 8B compares the average Jaccard statistic values between *segment_liftover* and *UCSC liftOver*, ordered by the length of samples on the *x*-axis (Supplementary Figure S5b). Overall we found that *segment_liftover* can preserve the width of intervals more accurately than *UCSC liftOver* on all histone modification samples (*P*< 0.001) (Figure 8B). This result may be expected since the algorithm of *segment_liftover* is integrity preserving. Meanwhile, results from *segment_liftover* are not significantly different from *UCSC liftOver* (*P* = 0.331) in TF ChIP-seq data. Among the TF ChIP-seq data, the width of liftover output is highly correlated with its corresponding alignment output, with a median of *r* = 1.00 (1.00–1.00), both when using *segment_liftover* and *UCSC liftOver*, except for ESR1 ChIP-seq which displayed a considerably low *r* = 0.22 using *UCSC liftOver*. However the discordance of this sample was not observed by Jaccard statistic. We found that the discordance was caused by an insertion into gapped-in-hg38 that increased the width of the liftover interval to 136 899 bp, whereas in the alignment output the region actually contained 20 binding sites, which ranged in size from 123–1867 bp (Figure 8C). When *segment_liftover* was used, the correlation coefficient was high (*r* = 1.00) because these input intervals cannot be converted since they violated the criteria of the tool (Figure 8D). Except for H3K9me3 (median *r* = 0.58) and H3K27me3 (median *r* = 0.72), the other ChIP-seq data types had a median Pearson *r* > 0.75 (Figure 8B). Together our data show that *segment_liftover* outperforms *UCSC liftOver* in interval conversion in terms of accuracy of the width of ChIP-Seq segments that are converted.

### Three-step guideline to improve the result of conversion of epigenome sequencing data

We have implemented a guideline to improve the results of conversion of epigenome sequencing data, namely, *Lifted* (https://github.com/phuoc362/Lifted). From the enrichment analyses described in previous sections, all the gapped regions that can cause corruption were gathered to build three annotation files, namely, gapped-in-hg19, gapped-in-both and gapped-in-hg38. To liftover from hg19 to hg38, first, we filter the input coordinates by gapped-in-hg19, gapped-in-both and blacklist. Second, the remaining coordinates are all ungapped on hg19, so the liftover can be safely performed at this stage.

In the third step, we propose two options to remove gapped-in-hg38. The first option is conservative where all output intervals that overlap with gapped-in-hg38 are excluded after liftover (Supplementary Figure S7a). This ensures that only the input data situated in the ungapped regions will be converted and the users can then maximize output quality. However this approach will result in a reduction of the amount of converted data but the output will be of high quality. The second option is less conservative, whereby the input intervals in hg19 that overlap the coordinates of gapped-in-hg38 are split before liftover to cut out 2bp (Supplementary Figure S7b). This approach can preserve the number of convertible intervals, however the quality of conversion may not be as high and will contain false positive. The users should consider the advantages and disadvantages of each option to choose the appropriate one for their purposes. In both approaches, the inappropriate data such as duplication, alternative chromosome and not CG is filtered.

However, still there is a rare but unavoidable issue should be noted. It is expected that the sequence accompanied by successfully converted coordinates has preserved the same sequence after liftover in the ungapped region. For example, input coordinates chr11:70798736–70798741 corresponding to the sequence AAGTTG from hg19 was converted into chr11:70952592–70952597 with the same sequence AAGTTG in hg38 (Figure 8E). However, when we converted the coordinates chr1:145176340–145176345 corresponding to sequence TGTGGC from hg19, the result we observed in hg38 was chr1:148712556–148712561 with a sequence GCCACA (Figure 8F). In hg19 this sequence is situated on the positive strand, but it was edited to be on the negative strand in hg38. Notably both of these examples are coordinates in the ungapped region, which is almost always successfully lifted, as shown in our results above. We found that a change in strand occurred in 0.41% of ungapped regions (Figure 8G). Therefore, liftover is reliable in coordinate conversion but the corresponding sequence to these coordinates may not be equivalent between the two assemblies. Users can use the coordinates from liftover to perform downstream analysis, but they should be aware that sequence in some circumstances does not always remain the same before and after liftover.

## DISCUSSION

The task of genome conversion between coordinates from different versions of sequencing data is a tedious but an essential task to enable integration, visualiszation and analyses of data from multiple sources. The practice of liftover between genome assemblies is a convenient approach but concerns have been raised about its accuracy in comparison to a more computationally demanding re-alignment approach (https://groups.google.com/a/soe.ucsc.edu/forum/#!topic/genome/5H3qJzXNfwE, https://groups.google.com/a/soe.ucsc.edu/forum/#!topic/genome/WDf4uGMg5jg). This is the first study that comprehensively evaluated six of the most commonly used liftover tools for epigenomic sequencing data analyses.

First, when considering the mechanism underlying the liftover approach we found that only the ungapped blocks are well converted to the corresponding regions and maintain their width. In contrast the gapped-in-hg19 and gapped-in-both most commonly fail to convert. Even if these sequences were converted, their output coordinates were likely to be situated in an inappropriate region of the newer genome build and in particular the gapped-in-hg38 blocks often were found to have substantial increases in their width.

Second, we found that the overall performance was similar between the six liftover tools we tested for conversion between hg19 and hg38 genome, with at least 99.21% of the data preserved after being converted and filtered. This is consistent with a previous benchmarking study by Fortier of an exome sequencing dataset, which showed similar performance of over 96.0% correctly mapped SNPs when comparing among *UCSC liftOver*, *NCBI Remap* and a specific tool for variant conversion called *GHI Liftover* (http://blog.goldenhelix.com/nfortier/bridging-two-worlds-lifting-variants-grch38/). While Fortier did not investigate the cause of unconvertible variants, based on our data, we suggest this could be due to the problematic gapped regions. The difference between the most efficient tool and the least efficient tool in our study was 0.20% in CpG WGBS point data and 0.30% in interval ChIP-Seq data. The *UCSC liftOver* tool is one of the most commonly used tools (14), but users can choose any of the other tools we tested to liftover WGBS data according to their available resources. For example the available input data format (*UCSC liftOver* requires BED, *flo* requires GFF and the rest support a variety of formats), or importantly the users familiar programming language which can differ between the tools (Table 1). Regarding the ChIP-Seq data, we suggest that the users consider implementing the integrity-preserved tools for intervals data (*NCBI Remap* and *segment_liftover*) as even though they generated less intervals, the quality of the output data was more accurate.

Third, for WGBS data we found that liftover can map features such as CpG sites to the correct location on the new assembly in the majority of locations. Moreover, we also found extremely high correlation coefficients for both coverage and DNA methylation profile between the liftover output and alignment output. In our previous study comparing the performance of two Illumina sequencing platforms, HiSeq 2500 and X Ten, the concordance was found to be *r* = 0.94–0.97 and kappa = 0.60–0.75 (3). Our new data therefore suggest that using liftover tools on WGBS data may be more reproducible than resequencing the same sample on a different sequencing platform. Strong concordance was also found between liftover and alignment DMRs between two different methlyomes also supporting the accuracy of liftover for these studies.

Fourth, for interval conversion, liftover can also preserve annotation data of the samples, as we found a high percentage of overlapping and equal size of intervals between liftover and alignment output. In a previous study by Yang *et al.* (33), agreement between pairs of replicates for TF ChIP-seq samples was supported with kappa value ranging from 0.80 to 0.90, while kappa between pairs of liftover and alignment outputs of TF samples in our study was over 0.99 (Supplementary Figure S6). Similar to WGBS data, we found that liftover is more reproducible than resequencing of ChIP-seq. Considering the performance of tools, *segment_liftover* outperformed *UCSC liftOver* due to its integrity-preserved algorithm. When ChIP-Seq samples were grouped according to the types of histone modifications or TF mode, we found that the average width of ChIP-seq peaks does not cause a great impact on the liftover performance. Rather the performance is influenced by the gapped or ungapped regions where the input coordinates are situated. For example if the protein binding sites or histone modification regions overlap any gapped regions, the liftover tool will either fail to convert or will inappropriately assign. Moreover, the data (Figure 8B) suggest that users should be cautious of converting H3K9me3 and H3K27me3. Noting that *segment_liftover* is a tool built on top of *UCSC liftOver* with some criteria to correct the results. The algorithm appeared to be effective since it can reject severely inaccurate conversion such as the ESR1 binding site in our data, however it still does not warrant for cases that require the results to be more reliable with criteria such as comparing the length of the new segment and old segment. The gapped regions, which has been found as the main cause of most conversion corruptions, should be excluded either before or after the liftover process to minimize errors.

Finally we have developed a three-step guideline to improve the result of conversion of epigenome sequencing data, namely, *Lifted* (https://github.com/phuoc362/Lifted), to allow for more accurate liftover of epigenome sequencing data that will also be applicable for the next genome build.

## CONCLUSION

Liftover is a rational solution for conversion between genome assemblies by coordinates when there is a lack of data storage, computing resource, time or human resource as it can reproduce highly accurate results for downstream analysis of epigenome sequencing data. However, it is worth noting that liftover can only assure the accuracy of coordinates and annotation conversion, therefore assumptions of achieving equivalent corresponding sequences genome-wide should not always be made. We clarify the principle underlying this powerful tool and propose a strategy to overcome the limitations of liftover at problematic regions.

## DATA AVAILABILITY

All track data of gapped-in-hg19, gapped-in-both, gapped-in-hg38, blacklist, not CG and all the scripts used in this study are available in github (https://github.com/phuoc362/Lifted).

## Supplementary Material

lqaa054_Supplemental_Files
