# Supplementary material for: Benchmark study comparing liftover tools for genome conversion of epigenome sequencing data
Source: NAR Genom Bioinform. 2020 Aug 6;2(3):lqaa054. doi: 10.1093/nargab/lqaa054 (PMC7671393; doi:10.1093/nargab/lqaa054)
Supplement: lqaa054_Supplemental_Files [file lqaa054_supplemental_files.zip › Supplementary_Figures_final.pdf]

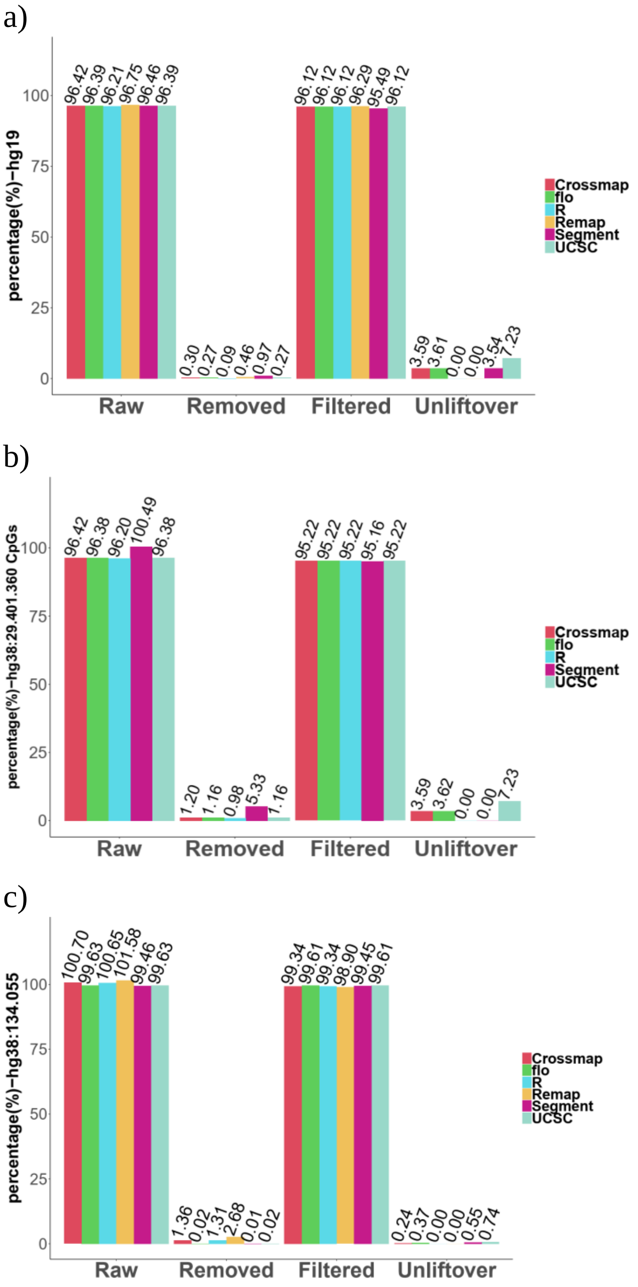

Supplementary Figure 1. Benchmarking 6 liftover tools from hg38 to hg19: *UCSC liftOver*, *rtracklayer::liftOver*, *CrossMap*, *NCBI Remap*, *flo*, and *segment_liftover*. **a)** Liftover 250,000 CpGs using 6 tools. **b)** Liftover of all CpGs using 5 tools (exclude *NCBI Remap*). **c)** Liftover of a ChIP-Seq sample comparing 6 tools.

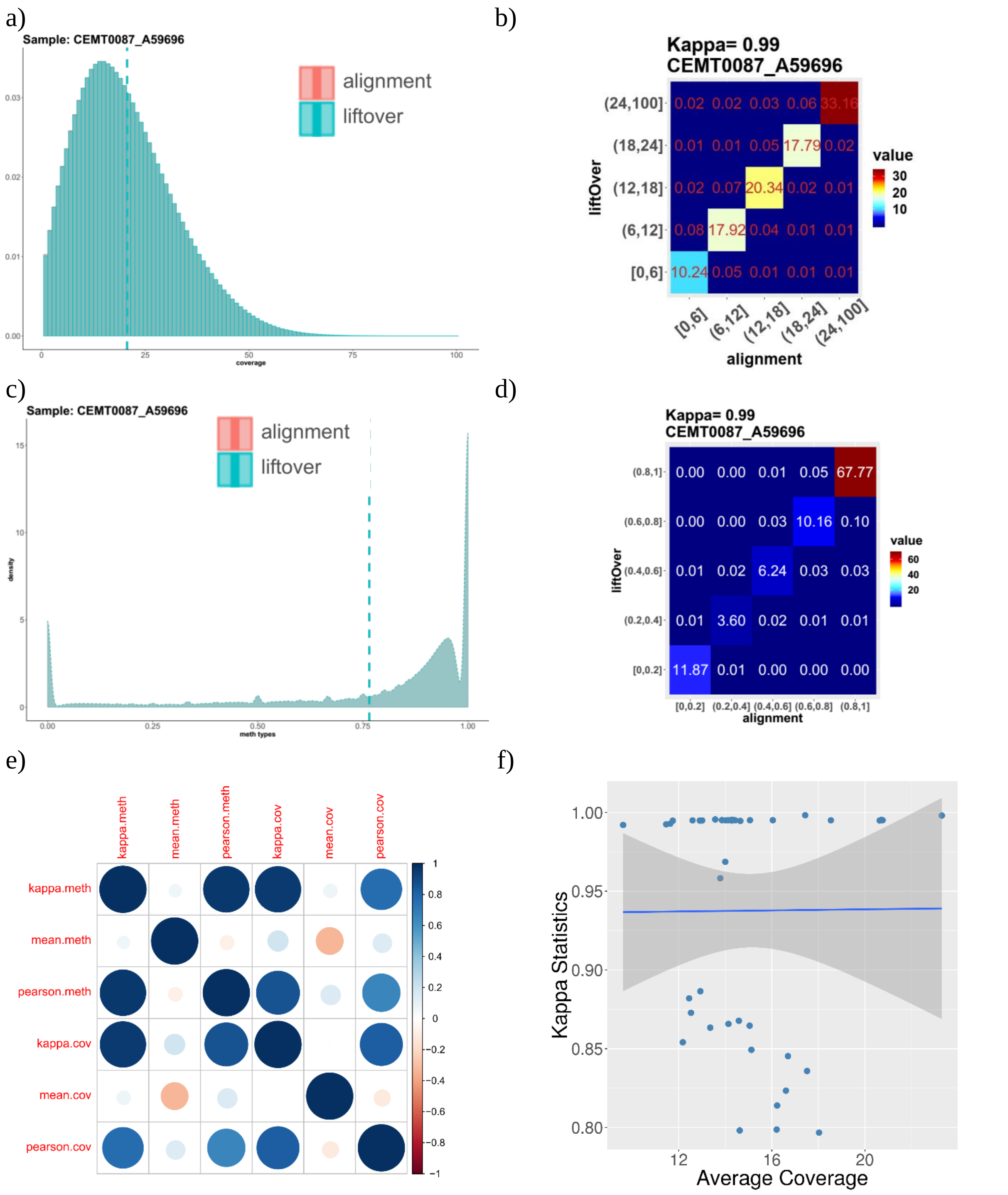

Supplementary Figure 2. a) Histogram showing distribution of coverage values of sample CEMT0087_A59696. **b)** Kappa statistic of coverage between *UCSC liftOver* and alignment output. **c)** Histogram showing distribution of DNA methylation values of sample CEMT0087_A59696. **d)** Kappa statistic of methylation between *UCSC liftOver* and alignment output. **e)** Correlation matrix among mean coverage, mean methylation value, kappa statistic and Pearson correlation. **f)** Scatter plot of the relationship between average coverage and kappa, with linear regression line.

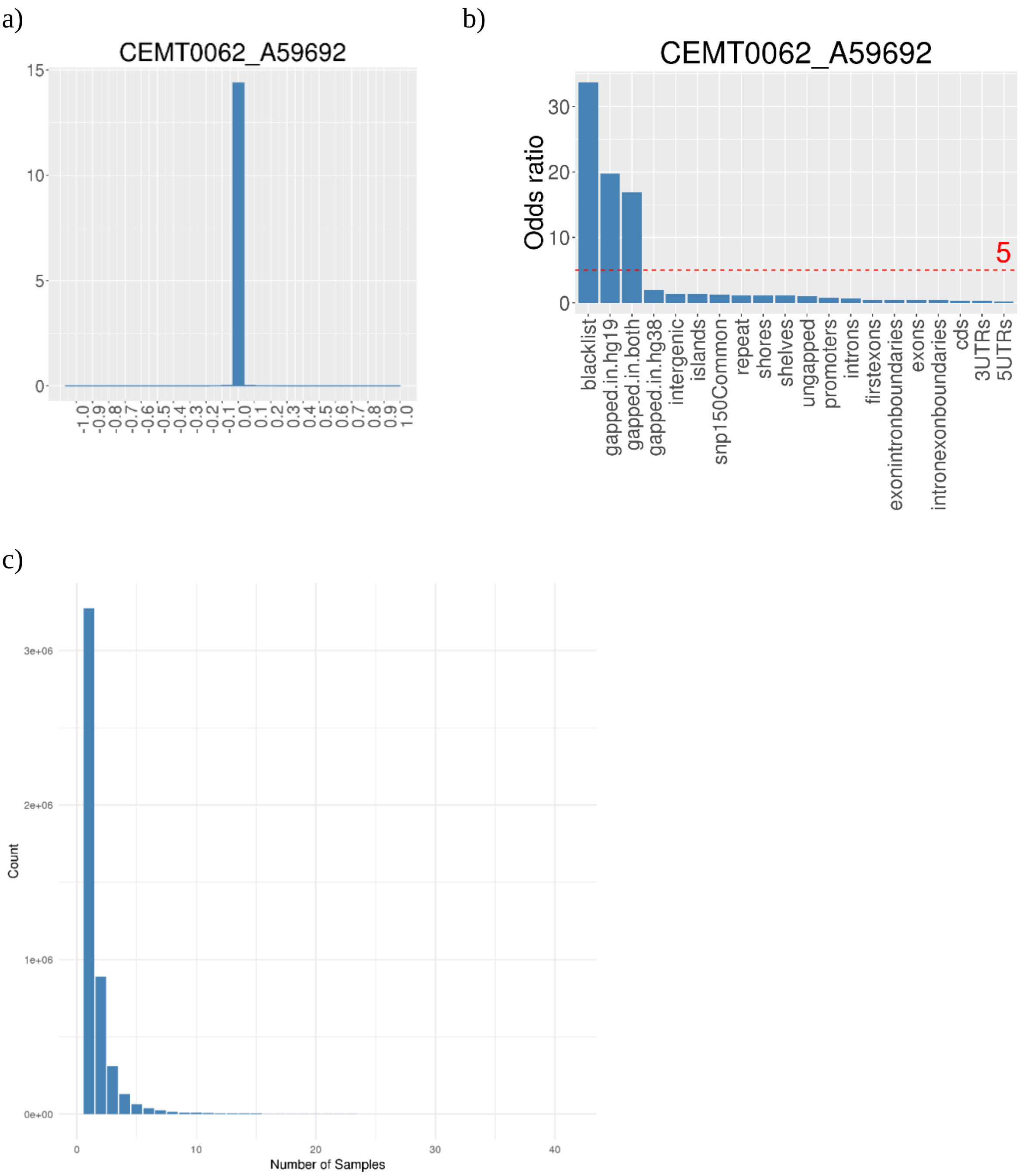

Supplementary Figure 3. DNA methylation analysis of WGBS. **a)** Distribution of delta values among CpGs in sample CEMT0087_A59696. **b)** Enrichment analysis of CpGs with delta ≥0.20 in sample CEMT0062_A59692. **c)** The number of occurrences of the same CpGs with delta ≥0.20 observed among 43 samples.

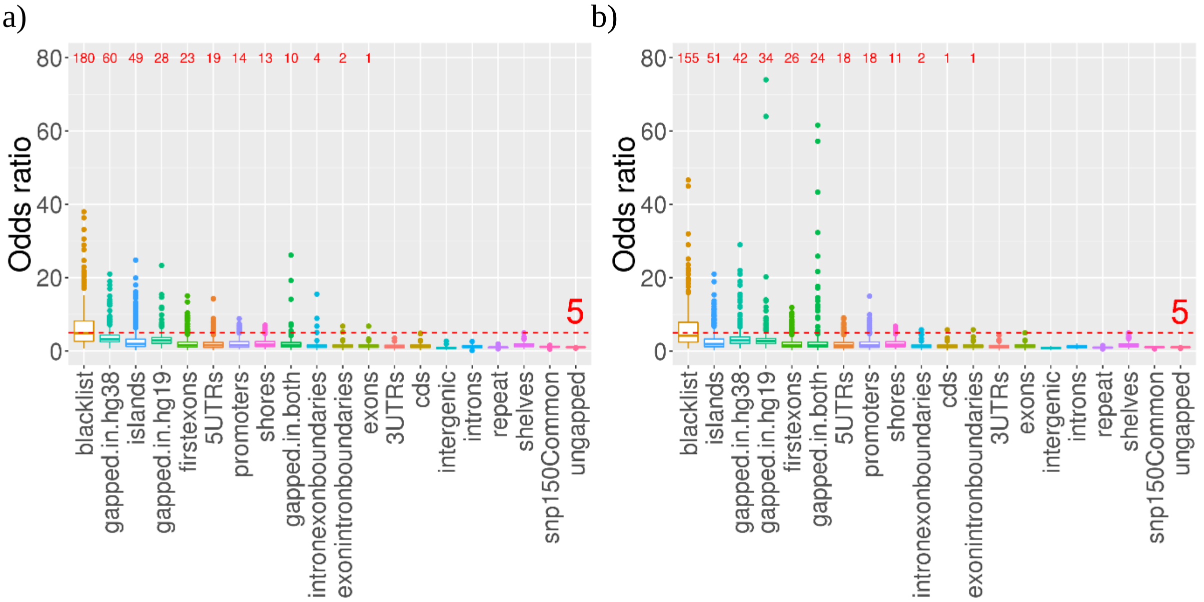

Supplementary Figure 4. a) Enrichment analysis of loss and gain intervals across 366 ChIP-Seq samples *UCSC liftOver* with 20 genomic features. **b)** Enrichment analysis of loss and gain intervals across 366 ChIP-Seq samples using *segment_liftover* with 20 genomic features.

a)

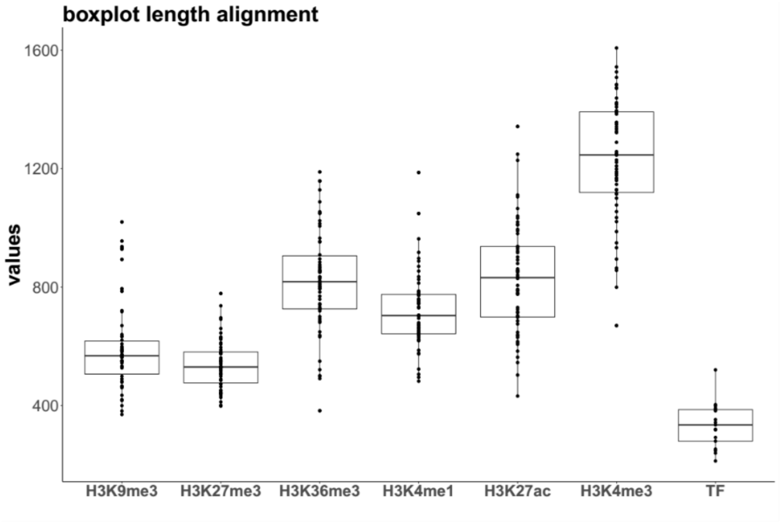

b)

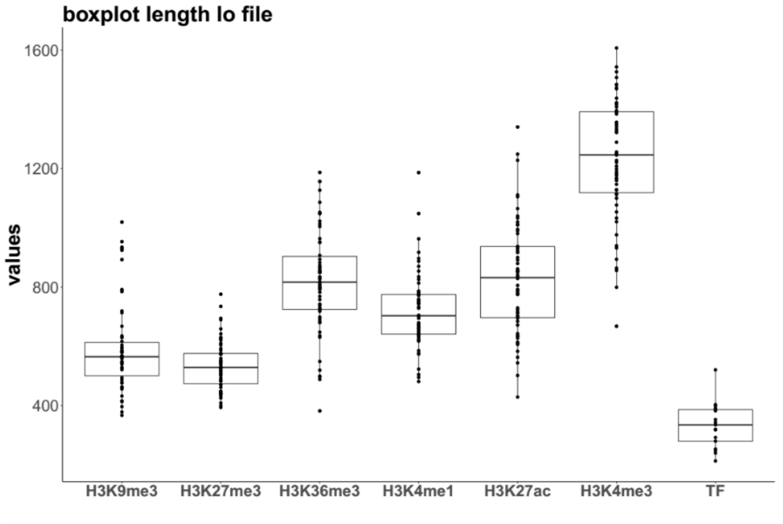

Supplementary Figure 5. a) Average lengths of 366 alignment outputs grouping by types of ChIP-Seq. **b)** Average lengths of 366 liftover outputs grouped by types of ChIP-Seq data.

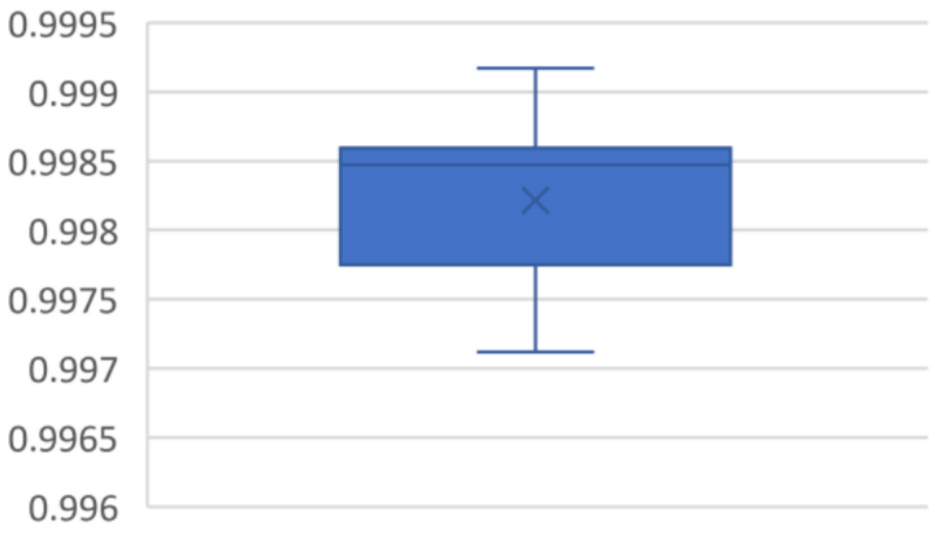

Supplementary Figure 6. Kappa values for agreement between liftover and alignment output of 18 TF samples.

a)

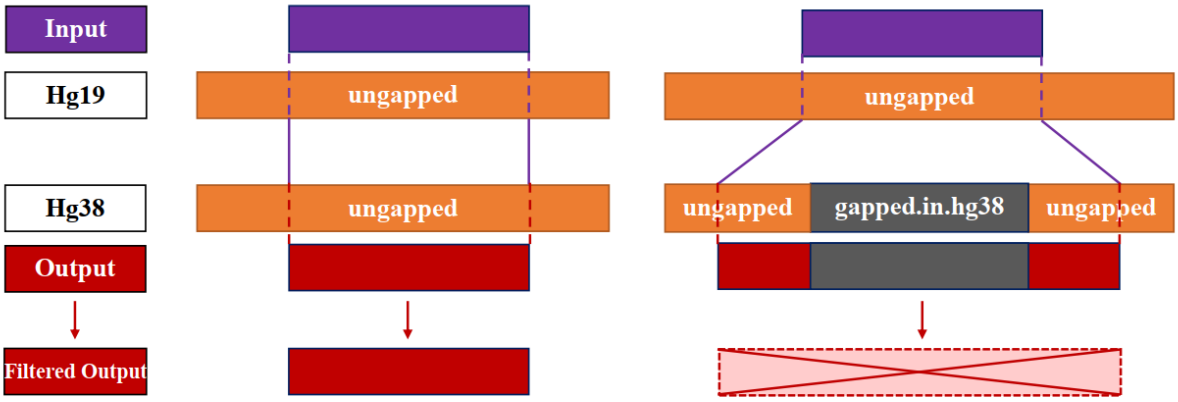

b)

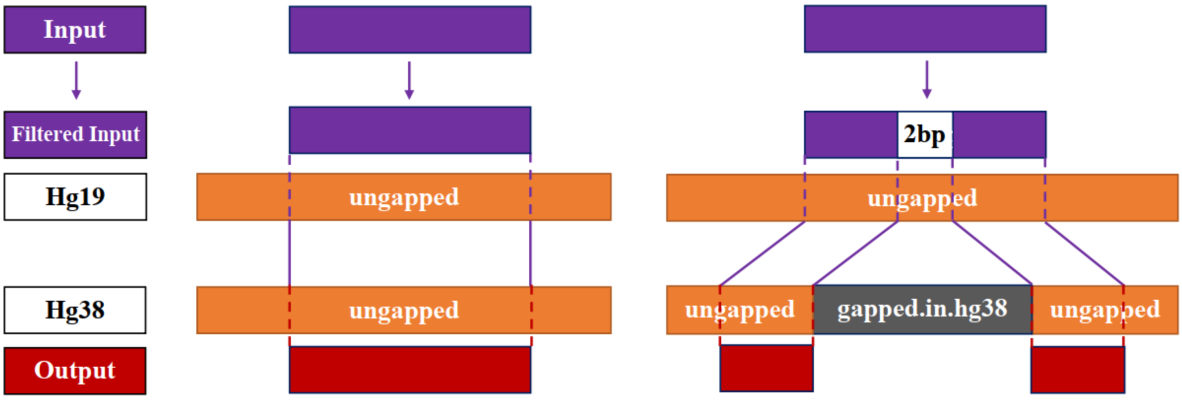

Supplementary Figure 7. Liftover with three-step *Lifted* guideline. **a)** Conservative *Lifted* (*cLifted*) which interval does not cross gapped-in-hg38 and interval crosses gapped-in-hg38. **b)** Less conservative *Lifted* (*lLifted*) which interval does not cross gapped-in-hg38 and interval crosses gapped-in-hg38
