# Supplementary material for: Benchmark study comparing liftover tools for genome conversion of epigenome sequencing data
Source: NAR Genom Bioinform. 2020 Aug 6;2(3):lqaa054. doi: 10.1093/nargab/lqaa054 (PMC7671393; doi:10.1093/nargab/lqaa054)
Supplement: lqaa054_Supplemental_Files [file lqaa054_supplemental_files.zip › Supplementary_Tables_final_corrected.pdf]

Supplementary Table 1. *Homo sapiens* reference genome

UCSC Version	Release Date	Release Name
hg38	Dec 2013	GRCh38
hg19	Feb 2009	GRCh37
hg18	Mar 2006	NCBI Build 36.1
hg17	May 2004	NCBI Build 35
hg16	Jul 2003	NCBI Build 34
hg15	Apr 2003	NCBI Build 33
hg13	Nov 2002	NCBI Build 31
hg12	Jun 2002	NCBI Build 30
…		
hg8	Aug 2001	UCSC assembled
…		
hg1	May 2000	UCSC assembled

GRC = Sanger, WashU, EBI and NCBI

Supplementary Table 2. List of 43 WGBS samples

Category		Name sample hg19	Library hg19	Sample name hg38	Library hg38	Short names of 100.000 CpGs
**Blood-Malignant**	*CD19+ cells (Chronic Lymphocytic Leukaemia)*	CEMT0004	A35999	CEMT0004	A35999	Bma1.g
	CD19+ cells (Chronic Lymphocytic Leukaemia)	CEMT0005	A36000	CEMT0005	A36000	Bma2.g
	CD19+ cells (Chronic Lymphocytic Leukaemia)	CEMT0006	A36001	CEMT0006	A36001	Bma3.g
	CD19+ cells (Chronic Lymphocytic Leukaemia)	CEMT0025	A36006	CEMT0025	A36006	Bma4.g
	CD19+ cells (Chronic Lymphocytic Leukaemia)	CEMT0026	A36315	CEMT0026	A36315	Bma5.g
	CD19+ cells (Chronic Lymphocytic Leukaemia)	CEMT0027	A36008	CEMT0027	A36008	Bma6.g
	CD19+ cells (Chronic Lymphocytic Leukaemia)	CEMT0028	A36009	CEMT0028	A36009	Bma7.g
	CD19+ cells (Chronic Lymphocytic Leukaemia)	CEMT0029	A36010	CEMT0029	A36010	Bma8.g
	CD19+ cells (Chronic Lymphocytic Leukaemia)	CEMT0030	A36011	CEMT0030	A36011	Bma9.g
	CD34+ cells(Chronic Myeloid Leukaemia)	CEMT0031	A50423-A50425	CEMT0031	A50423	Bma10.g
**Blood-Normal**	*CD34+ cells*	CEMT0032	A34042	CEMT0032	A34042	Bno1.g
**Colorectal-Normal**		CEMT0033	A50426-A50428	CEMT0033	A50428	Cno1.g
		CEMT0034	A50429-A50431	CEMT0034	A50431	Cno2.g
		CEMT0050	A54763	CEMT0050	A54763	Cno3.g
		CEMT0051	A54764	CEMT0051	A54764	Cno5.g
		CEMT0052	A54765	CEMT0052	A54765	Cno6.g
		CEMT0053	A54766	CEMT0053	A54766	Cno7.g

		CEMT0054	A54767	CEMT0054	A54767	Cno8.g
		CEMT0055	A54768	CEMT0055	A54768	Cno9.g
		CEMT0056	A54769	CEMT0056	A54769	Cno10.g
		CEMT0057	A54770	CEMT0057	A54770	Cno11.g
		CEMT0058	A54771	CEMT0058	A54771	Cno12.g
		CEMT0059	A54772	CEMT0059	A54772	Cno13.g
		CEMT0060	A54773	CEMT0060	A54773	Cno14.g
		CEMT0061	A54774	CEMT0061	A54774	Cno15.g
		CEMT0072	A59694	CEMT0072	A59694	Cno16.g
**Colorectal-Diseased**	*Malignant*	CEMT0062	A59692	CEMT0062	A59692	Cdi1.g
	Malignant	CEMT0063	A54775	CEMT0063	A54775	Cd2.g
	Malignant	CEMT0064	A54776	CEMT0064	A54776	Cdi3.g
	Malignant	CEMT0065	A54777	CEMT0065	A54777	Cdi4.g
	Malignant	CEMT0066	A54778	CEMT0066	A54778	Cdi5.g
	Malignant	CEMT0067	A54779	CEMT0067	A54779	Cdi6.g
	Diverticulisis	CEMT0068	A59693	CEMT0068	A59693	Cdi7.g
**Thyroid-Diseased**	*Adenoma-Follicular*	CEMT0041	A36013	CEMT0041	A36013	Tdi8.g
	Adenoma-Follicular	CEMT0043	A36015	CEMT0043	A36015	Tdi1.g
	Goiter	CEMT0045	A36017	CEMT0045	A36017	Tdi2.g
	Papillary Carcinoma	CEMT0087	A59696	CEMT0087	A59696	Tdi3.g
**Thyroid-Normal**		CEMT0040	A36012	CEMT0040	A36012	Tno1.g
		CEMT0042	A36014	CEMT0042	A36014	Tno3.g
		CEMT0044	A36016	CEMT0044	A36016	Tno4.g
		CEMT0086	A59695	CEMT0086	A59695	Tno5.g
**Cancer**	*LNCaP*	LNCaP		LNCaP		LNCaP.g
**Nomal**	*PrEC*	PrEC		PrEC		PrEC.g

Supplementary Table 3. List of 366 ChIP-Seq samples

Assay	Sample	Library	Samples name files hg19	Samples name files hg38
Histone	H3K27ac	CEMT0004	H3K27ac_CEMT0004.38.bw	H3K27ac_CEMT0004.19.bw
Histone	H3K27ac	CEMT0005	H3K27ac_CEMT0005.38.bw	H3K27ac_CEMT0005.19.bw
Histone	H3K27ac	CEMT0006	H3K27ac_CEMT0006.38.bw	H3K27ac_CEMT0006.19.bw
Histone	H3K27ac	CEMT0007	H3K27ac_CEMT0007.38.bw	H3K27ac_CEMT0007.19.bw
Histone	H3K27ac	CEMT0008	H3K27ac_CEMT0008.38.bw	H3K27ac_CEMT0008.19.bw
Histone	H3K27ac	CEMT0009	H3K27ac_CEMT0009.38.bw	H3K27ac_CEMT0009.19.bw
Histone	H3K27ac	CEMT0019	H3K27ac_CEMT0019.38.bw	H3K27ac_CEMT0019.19.bw
Histone	H3K27ac	CEMT0021	H3K27ac_CEMT0021.38.bw	H3K27ac_CEMT0021.19.bw
Histone	H3K27ac	CEMT0022	H3K27ac_CEMT0022.38.bw	H3K27ac_CEMT0022.19.bw
Histone	H3K27ac	CEMT0023	H3K27ac_CEMT0023.38.bw	H3K27ac_CEMT0023.19.bw
Histone	H3K27ac	CEMT0025	H3K27ac_CEMT0025.38.bw	H3K27ac_CEMT0025.19.bw
Histone	H3K27ac	CEMT0026	H3K27ac_CEMT0026.38.bw	H3K27ac_CEMT0026.19.bw

Histone	H3K27ac	CEMT0027	H3K27ac_CEMT0027.38.bw	H3K27ac_CEMT0027.19.bw
Histone	H3K27ac	CEMT0028	H3K27ac_CEMT0028.38.bw	H3K27ac_CEMT0028.19.bw
Histone	H3K27ac	CEMT0029	H3K27ac_CEMT0029.38.bw	H3K27ac_CEMT0029.19.bw
Histone	H3K27ac	CEMT0030	H3K27ac_CEMT0030.38.bw	H3K27ac_CEMT0030.19.bw
Histone	H3K27ac	CEMT0031	H3K27ac_CEMT0031.38.bw	H3K27ac_CEMT0031.19.bw
Histone	H3K27ac	CEMT0032	H3K27ac_CEMT0032.38.bw	H3K27ac_CEMT0032.19.bw
Histone	H3K27ac	CEMT0033	H3K27ac_CEMT0033.38.bw	H3K27ac_CEMT0033.19.bw
Histone	H3K27ac	CEMT0034	H3K27ac_CEMT0034.38.bw	H3K27ac_CEMT0034.19.bw
Histone	H3K27ac	CEMT0035	H3K27ac_CEMT0035.38.bw	H3K27ac_CEMT0035.19.bw
Histone	H3K27ac	CEMT0036	H3K27ac_CEMT0036.38.bw	H3K27ac_CEMT0036.19.bw
Histone	H3K27ac	CEMT0037	H3K27ac_CEMT0037.38.bw	H3K27ac_CEMT0037.19.bw
Histone	H3K27ac	CEMT0038	H3K27ac_CEMT0038.38.bw	H3K27ac_CEMT0038.19.bw
Histone	H3K27ac	CEMT0047	H3K27ac_CEMT0047.38.bw	H3K27ac_CEMT0047.19.bw
Histone	H3K27ac	CEMT0050	H3K27ac_CEMT0050.38.bw	H3K27ac_CEMT0050.19.bw
Histone	H3K27ac	CEMT0051	H3K27ac_CEMT0051.38.bw	H3K27ac_CEMT0051.19.bw
Histone	H3K27ac	CEMT0052	H3K27ac_CEMT0052.38.bw	H3K27ac_CEMT0052.19.bw
Histone	H3K27ac	CEMT0053	H3K27ac_CEMT0053.38.bw	H3K27ac_CEMT0053.19.bw
Histone	H3K27ac	CEMT0054	H3K27ac_CEMT0054.38.bw	H3K27ac_CEMT0054.19.bw
Histone	H3K27ac	CEMT0055	H3K27ac_CEMT0055.38.bw	H3K27ac_CEMT0055.19.bw
Histone	H3K27ac	CEMT0056	H3K27ac_CEMT0056.38.bw	H3K27ac_CEMT0056.19.bw
Histone	H3K27ac	CEMT0057	H3K27ac_CEMT0057.38.bw	H3K27ac_CEMT0057.19.bw
Histone	H3K27ac	CEMT0058	H3K27ac_CEMT0058.38.bw	H3K27ac_CEMT0058.19.bw
Histone	H3K27ac	CEMT0059	H3K27ac_CEMT0059.38.bw	H3K27ac_CEMT0059.19.bw
Histone	H3K27ac	CEMT0060	H3K27ac_CEMT0060.38.bw	H3K27ac_CEMT0060.19.bw
Histone	H3K27ac	CEMT0061	H3K27ac_CEMT0061.38.bw	H3K27ac_CEMT0061.19.bw
Histone	H3K27ac	CEMT0062	H3K27ac_CEMT0062.38.bw	H3K27ac_CEMT0062.19.bw
Histone	H3K27ac	CEMT0063	H3K27ac_CEMT0063.38.bw	H3K27ac_CEMT0063.19.bw
Histone	H3K27ac	CEMT0064	H3K27ac_CEMT0064.38.bw	H3K27ac_CEMT0064.19.bw
Histone	H3K27ac	CEMT0065	H3K27ac_CEMT0065.38.bw	H3K27ac_CEMT0065.19.bw
Histone	H3K27ac	CEMT0066	H3K27ac_CEMT0066.38.bw	H3K27ac_CEMT0066.19.bw
Histone	H3K27ac	CEMT0067	H3K27ac_CEMT0067.38.bw	H3K27ac_CEMT0067.19.bw
Histone	H3K27ac	CEMT0068	H3K27ac_CEMT0068.38.bw	H3K27ac_CEMT0068.19.bw
Histone	H3K27ac	CEMT0072	H3K27ac_CEMT0072.38.bw	H3K27ac_CEMT0072.19.bw
Histone	H3K27ac	CEMT0073	H3K27ac_CEMT0073.38.bw	H3K27ac_CEMT0073.19.bw
Histone	H3K27ac	CEMT0074	H3K27ac_CEMT0074.38.bw	H3K27ac_CEMT0074.19.bw
Histone	H3K27ac	CEMT0075	H3K27ac_CEMT0075.38.bw	H3K27ac_CEMT0075.19.bw
Histone	H3K27ac	CEMT0076	H3K27ac_CEMT0076.38.bw	H3K27ac_CEMT0076.19.bw
Histone	H3K27ac	CEMT0078	H3K27ac_CEMT0078.38.bw	H3K27ac_CEMT0078.19.bw
Histone	H3K27ac	CEMT0079	H3K27ac_CEMT0079.38.bw	H3K27ac_CEMT0079.19.bw
Histone	H3K27ac	CEMT0081	H3K27ac_CEMT0081.38.bw	H3K27ac_CEMT0081.19.bw
Histone	H3K27ac	CEMT0094	H3K27ac_CEMT0094.38.bw	H3K27ac_CEMT0094.19.bw
Histone	H3K27ac	CEMT0095	H3K27ac_CEMT0095.38.bw	H3K27ac_CEMT0095.19.bw

Histone	H3K27ac	CEMT0096	H3K27ac_CEMT0096.38.bw	H3K27ac_CEMT0096.19.bw
Histone	H3K27ac	CEMT0097	H3K27ac_CEMT0097.38.bw	H3K27ac_CEMT0097.19.bw
Histone	H3K27ac	CEMT0149	H3K27ac_CEMT0149.38.bw	H3K27ac_CEMT0149.19.bw
Histone	H3K27ac	CEMT0150	H3K27ac_CEMT0150.38.bw	H3K27ac_CEMT0150.19.bw
Histone	H3K27me3	CEMT0004	H3K27me3_CEMT0004.38.bw	H3K27me3_CEMT0004.19.bw
Histone	H3K27me3	CEMT0005	H3K27me3_CEMT0005.38.bw	H3K27me3_CEMT0005.19.bw
Histone	H3K27me3	CEMT0006	H3K27me3_CEMT0006.38.bw	H3K27me3_CEMT0006.19.bw
Histone	H3K27me3	CEMT0007	H3K27me3_CEMT0007.38.bw	H3K27me3_CEMT0007.19.bw
Histone	H3K27me3	CEMT0008	H3K27me3_CEMT0008.38.bw	H3K27me3_CEMT0008.19.bw
Histone	H3K27me3	CEMT0009	H3K27me3_CEMT0009.38.bw	H3K27me3_CEMT0009.19.bw
Histone	H3K27me3	CEMT0019	H3K27me3_CEMT0019.38.bw	H3K27me3_CEMT0019.19.bw
Histone	H3K27me3	CEMT0021	H3K27me3_CEMT0021.38.bw	H3K27me3_CEMT0021.19.bw
Histone	H3K27me3	CEMT0022	H3K27me3_CEMT0022.38.bw	H3K27me3_CEMT0022.19.bw
Histone	H3K27me3	CEMT0023	H3K27me3_CEMT0023.38.bw	H3K27me3_CEMT0023.19.bw
Histone	H3K27me3	CEMT0025	H3K27me3_CEMT0025.38.bw	H3K27me3_CEMT0025.19.bw
Histone	H3K27me3	CEMT0026	H3K27me3_CEMT0026.38.bw	H3K27me3_CEMT0026.19.bw
Histone	H3K27me3	CEMT0027	H3K27me3_CEMT0027.38.bw	H3K27me3_CEMT0027.19.bw
Histone	H3K27me3	CEMT0028	H3K27me3_CEMT0028.38.bw	H3K27me3_CEMT0028.19.bw
Histone	H3K27me3	CEMT0029	H3K27me3_CEMT0029.38.bw	H3K27me3_CEMT0029.19.bw
Histone	H3K27me3	CEMT0030	H3K27me3_CEMT0030.38.bw	H3K27me3_CEMT0030.19.bw
Histone	H3K27me3	CEMT0031	H3K27me3_CEMT0031.38.bw	H3K27me3_CEMT0031.19.bw
Histone	H3K27me3	CEMT0032	H3K27me3_CEMT0032.38.bw	H3K27me3_CEMT0032.19.bw
Histone	H3K27me3	CEMT0033	H3K27me3_CEMT0033.38.bw	H3K27me3_CEMT0033.19.bw
Histone	H3K27me3	CEMT0034	H3K27me3_CEMT0034.38.bw	H3K27me3_CEMT0034.19.bw
Histone	H3K27me3	CEMT0035	H3K27me3_CEMT0035.38.bw	H3K27me3_CEMT0035.19.bw
Histone	H3K27me3	CEMT0036	H3K27me3_CEMT0036.38.bw	H3K27me3_CEMT0036.19.bw
Histone	H3K27me3	CEMT0037	H3K27me3_CEMT0037.38.bw	H3K27me3_CEMT0037.19.bw
Histone	H3K27me3	CEMT0038	H3K27me3_CEMT0038.38.bw	H3K27me3_CEMT0038.19.bw
Histone	H3K27me3	CEMT0047	H3K27me3_CEMT0047.38.bw	H3K27me3_CEMT0047.19.bw
Histone	H3K27me3	CEMT0050	H3K27me3_CEMT0050.38.bw	H3K27me3_CEMT0050.19.bw
Histone	H3K27me3	CEMT0051	H3K27me3_CEMT0051.38.bw	H3K27me3_CEMT0051.19.bw
Histone	H3K27me3	CEMT0052	H3K27me3_CEMT0052.38.bw	H3K27me3_CEMT0052.19.bw
Histone	H3K27me3	CEMT0053	H3K27me3_CEMT0053.38.bw	H3K27me3_CEMT0053.19.bw
Histone	H3K27me3	CEMT0054	H3K27me3_CEMT0054.38.bw	H3K27me3_CEMT0054.19.bw
Histone	H3K27me3	CEMT0055	H3K27me3_CEMT0055.38.bw	H3K27me3_CEMT0055.19.bw
Histone	H3K27me3	CEMT0056	H3K27me3_CEMT0056.38.bw	H3K27me3_CEMT0056.19.bw
Histone	H3K27me3	CEMT0057	H3K27me3_CEMT0057.38.bw	H3K27me3_CEMT0057.19.bw
Histone	H3K27me3	CEMT0058	H3K27me3_CEMT0058.38.bw	H3K27me3_CEMT0058.19.bw
Histone	H3K27me3	CEMT0059	H3K27me3_CEMT0059.38.bw	H3K27me3_CEMT0059.19.bw
Histone	H3K27me3	CEMT0060	H3K27me3_CEMT0060.38.bw	H3K27me3_CEMT0060.19.bw
Histone	H3K27me3	CEMT0061	H3K27me3_CEMT0061.38.bw	H3K27me3_CEMT0061.19.bw
Histone	H3K27me3	CEMT0062	H3K27me3_CEMT0062.38.bw	H3K27me3_CEMT0062.19.bw

Histone	H3K27me3	CEMT0063	H3K27me3_CEMT0063.38.bw	H3K27me3_CEMT0063.19.bw
Histone	H3K27me3	CEMT0064	H3K27me3_CEMT0064.38.bw	H3K27me3_CEMT0064.19.bw
Histone	H3K27me3	CEMT0065	H3K27me3_CEMT0065.38.bw	H3K27me3_CEMT0065.19.bw
Histone	H3K27me3	CEMT0066	H3K27me3_CEMT0066.38.bw	H3K27me3_CEMT0066.19.bw
Histone	H3K27me3	CEMT0067	H3K27me3_CEMT0067.38.bw	H3K27me3_CEMT0067.19.bw
Histone	H3K27me3	CEMT0068	H3K27me3_CEMT0068.38.bw	H3K27me3_CEMT0068.19.bw
Histone	H3K27me3	CEMT0072	H3K27me3_CEMT0072.38.bw	H3K27me3_CEMT0072.19.bw
Histone	H3K27me3	CEMT0073	H3K27me3_CEMT0073.38.bw	H3K27me3_CEMT0073.19.bw
Histone	H3K27me3	CEMT0074	H3K27me3_CEMT0074.38.bw	H3K27me3_CEMT0074.19.bw
Histone	H3K27me3	CEMT0075	H3K27me3_CEMT0075.38.bw	H3K27me3_CEMT0075.19.bw
Histone	H3K27me3	CEMT0076	H3K27me3_CEMT0076.38.bw	H3K27me3_CEMT0076.19.bw
Histone	H3K27me3	CEMT0078	H3K27me3_CEMT0078.38.bw	H3K27me3_CEMT0078.19.bw
Histone	H3K27me3	CEMT0079	H3K27me3_CEMT0079.38.bw	H3K27me3_CEMT0079.19.bw
Histone	H3K27me3	CEMT0081	H3K27me3_CEMT0081.38.bw	H3K27me3_CEMT0081.19.bw
Histone	H3K27me3	CEMT0094	H3K27me3_CEMT0094.38.bw	H3K27me3_CEMT0094.19.bw
Histone	H3K27me3	CEMT0095	H3K27me3_CEMT0095.38.bw	H3K27me3_CEMT0095.19.bw
Histone	H3K27me3	CEMT0096	H3K27me3_CEMT0096.38.bw	H3K27me3_CEMT0096.19.bw
Histone	H3K27me3	CEMT0097	H3K27me3_CEMT0097.38.bw	H3K27me3_CEMT0097.19.bw
Histone	H3K27me3	CEMT0149	H3K27me3_CEMT0149.38.bw	H3K27me3_CEMT0149.19.bw
Histone	H3K27me3	CEMT0150	H3K27me3_CEMT0150.38.bw	H3K27me3_CEMT0150.19.bw
Histone	H3K36me3	CEMT0004	H3K36me3_CEMT0004.38.bw	H3K36me3_CEMT0004.19.bw
Histone	H3K36me3	CEMT0005	H3K36me3_CEMT0005.38.bw	H3K36me3_CEMT0005.19.bw
Histone	H3K36me3	CEMT0006	H3K36me3_CEMT0006.38.bw	H3K36me3_CEMT0006.19.bw
Histone	H3K36me3	CEMT0007	H3K36me3_CEMT0007.38.bw	H3K36me3_CEMT0007.19.bw
Histone	H3K36me3	CEMT0008	H3K36me3_CEMT0008.38.bw	H3K36me3_CEMT0008.19.bw
Histone	H3K36me3	CEMT0009	H3K36me3_CEMT0009.38.bw	H3K36me3_CEMT0009.19.bw
Histone	H3K36me3	CEMT0019	H3K36me3_CEMT0019.38.bw	H3K36me3_CEMT0019.19.bw
Histone	H3K36me3	CEMT0021	H3K36me3_CEMT0021.38.bw	H3K36me3_CEMT0021.19.bw
Histone	H3K36me3	CEMT0022	H3K36me3_CEMT0022.38.bw	H3K36me3_CEMT0022.19.bw
Histone	H3K36me3	CEMT0023	H3K36me3_CEMT0023.38.bw	H3K36me3_CEMT0023.19.bw
Histone	H3K36me3	CEMT0025	H3K36me3_CEMT0025.38.bw	H3K36me3_CEMT0025.19.bw
Histone	H3K36me3	CEMT0026	H3K36me3_CEMT0026.38.bw	H3K36me3_CEMT0026.19.bw
Histone	H3K36me3	CEMT0027	H3K36me3_CEMT0027.38.bw	H3K36me3_CEMT0027.19.bw
Histone	H3K36me3	CEMT0028	H3K36me3_CEMT0028.38.bw	H3K36me3_CEMT0028.19.bw
Histone	H3K36me3	CEMT0029	H3K36me3_CEMT0029.38.bw	H3K36me3_CEMT0029.19.bw
Histone	H3K36me3	CEMT0030	H3K36me3_CEMT0030.38.bw	H3K36me3_CEMT0030.19.bw
Histone	H3K36me3	CEMT0031	H3K36me3_CEMT0031.38.bw	H3K36me3_CEMT0031.19.bw
Histone	H3K36me3	CEMT0032	H3K36me3_CEMT0032.38.bw	H3K36me3_CEMT0032.19.bw
Histone	H3K36me3	CEMT0033	H3K36me3_CEMT0033.38.bw	H3K36me3_CEMT0033.19.bw
Histone	H3K36me3	CEMT0034	H3K36me3_CEMT0034.38.bw	H3K36me3_CEMT0034.19.bw
Histone	H3K36me3	CEMT0035	H3K36me3_CEMT0035.38.bw	H3K36me3_CEMT0035.19.bw
Histone	H3K36me3	CEMT0036	H3K36me3_CEMT0036.38.bw	H3K36me3_CEMT0036.19.bw

Histone	H3K36me3	CEMT0037	H3K36me3_CEMT0037.38.bw	H3K36me3_CEMT0037.19.bw
Histone	H3K36me3	CEMT0038	H3K36me3_CEMT0038.38.bw	H3K36me3_CEMT0038.19.bw
Histone	H3K36me3	CEMT0047	H3K36me3_CEMT0047.38.bw	H3K36me3_CEMT0047.19.bw
Histone	H3K36me3	CEMT0050	H3K36me3_CEMT0050.38.bw	H3K36me3_CEMT0050.19.bw
Histone	H3K36me3	CEMT0051	H3K36me3_CEMT0051.38.bw	H3K36me3_CEMT0051.19.bw
Histone	H3K36me3	CEMT0052	H3K36me3_CEMT0052.38.bw	H3K36me3_CEMT0052.19.bw
Histone	H3K36me3	CEMT0053	H3K36me3_CEMT0053.38.bw	H3K36me3_CEMT0053.19.bw
Histone	H3K36me3	CEMT0054	H3K36me3_CEMT0054.38.bw	H3K36me3_CEMT0054.19.bw
Histone	H3K36me3	CEMT0055	H3K36me3_CEMT0055.38.bw	H3K36me3_CEMT0055.19.bw
Histone	H3K36me3	CEMT0056	H3K36me3_CEMT0056.38.bw	H3K36me3_CEMT0056.19.bw
Histone	H3K36me3	CEMT0057	H3K36me3_CEMT0057.38.bw	H3K36me3_CEMT0057.19.bw
Histone	H3K36me3	CEMT0058	H3K36me3_CEMT0058.38.bw	H3K36me3_CEMT0058.19.bw
Histone	H3K36me3	CEMT0059	H3K36me3_CEMT0059.38.bw	H3K36me3_CEMT0059.19.bw
Histone	H3K36me3	CEMT0060	H3K36me3_CEMT0060.38.bw	H3K36me3_CEMT0060.19.bw
Histone	H3K36me3	CEMT0061	H3K36me3_CEMT0061.38.bw	H3K36me3_CEMT0061.19.bw
Histone	H3K36me3	CEMT0062	H3K36me3_CEMT0062.38.bw	H3K36me3_CEMT0062.19.bw
Histone	H3K36me3	CEMT0063	H3K36me3_CEMT0063.38.bw	H3K36me3_CEMT0063.19.bw
Histone	H3K36me3	CEMT0064	H3K36me3_CEMT0064.38.bw	H3K36me3_CEMT0064.19.bw
Histone	H3K36me3	CEMT0065	H3K36me3_CEMT0065.38.bw	H3K36me3_CEMT0065.19.bw
Histone	H3K36me3	CEMT0066	H3K36me3_CEMT0066.38.bw	H3K36me3_CEMT0066.19.bw
Histone	H3K36me3	CEMT0067	H3K36me3_CEMT0067.38.bw	H3K36me3_CEMT0067.19.bw
Histone	H3K36me3	CEMT0068	H3K36me3_CEMT0068.38.bw	H3K36me3_CEMT0068.19.bw
Histone	H3K36me3	CEMT0072	H3K36me3_CEMT0072.38.bw	H3K36me3_CEMT0072.19.bw
Histone	H3K36me3	CEMT0073	H3K36me3_CEMT0073.38.bw	H3K36me3_CEMT0073.19.bw
Histone	H3K36me3	CEMT0074	H3K36me3_CEMT0074.38.bw	H3K36me3_CEMT0074.19.bw
Histone	H3K36me3	CEMT0075	H3K36me3_CEMT0075.38.bw	H3K36me3_CEMT0075.19.bw
Histone	H3K36me3	CEMT0076	H3K36me3_CEMT0076.38.bw	H3K36me3_CEMT0076.19.bw
Histone	H3K36me3	CEMT0078	H3K36me3_CEMT0078.38.bw	H3K36me3_CEMT0078.19.bw
Histone	H3K36me3	CEMT0079	H3K36me3_CEMT0079.38.bw	H3K36me3_CEMT0079.19.bw
Histone	H3K36me3	CEMT0081	H3K36me3_CEMT0081.38.bw	H3K36me3_CEMT0081.19.bw
Histone	H3K36me3	CEMT0094	H3K36me3_CEMT0094.38.bw	H3K36me3_CEMT0094.19.bw
Histone	H3K36me3	CEMT0095	H3K36me3_CEMT0095.38.bw	H3K36me3_CEMT0095.19.bw
Histone	H3K36me3	CEMT0096	H3K36me3_CEMT0096.38.bw	H3K36me3_CEMT0096.19.bw
Histone	H3K36me3	CEMT0097	H3K36me3_CEMT0097.38.bw	H3K36me3_CEMT0097.19.bw
Histone	H3K36me3	CEMT0149	H3K36me3_CEMT0149.38.bw	H3K36me3_CEMT0149.19.bw
Histone	H3K36me3	CEMT0150	H3K36me3_CEMT0150.38.bw	H3K36me3_CEMT0150.19.bw
Histone	H3K4me1	CEMT0004	H3K4me1_CEMT0004.38.bw	H3K4me1_CEMT0004.19.bw
Histone	H3K4me1	CEMT0005	H3K4me1_CEMT0005.38.bw	H3K4me1_CEMT0005.19.bw
Histone	H3K4me1	CEMT0006	H3K4me1_CEMT0006.38.bw	H3K4me1_CEMT0006.19.bw
Histone	H3K4me1	CEMT0007	H3K4me1_CEMT0007.38.bw	H3K4me1_CEMT0007.19.bw
Histone	H3K4me1	CEMT0008	H3K4me1_CEMT0008.38.bw	H3K4me1_CEMT0008.19.bw
Histone	H3K4me1	CEMT0009	H3K4me1_CEMT0009.38.bw	H3K4me1_CEMT0009.19.bw

Histone	H3K4me1	CEMT0019	H3K4me1_CEMT0019.38.bw	H3K4me1_CEMT0019.19.bw
Histone	H3K4me1	CEMT0021	H3K4me1_CEMT0021.38.bw	H3K4me1_CEMT0021.19.bw
Histone	H3K4me1	CEMT0022	H3K4me1_CEMT0022.38.bw	H3K4me1_CEMT0022.19.bw
Histone	H3K4me1	CEMT0023	H3K4me1_CEMT0023.38.bw	H3K4me1_CEMT0023.19.bw
Histone	H3K4me1	CEMT0025	H3K4me1_CEMT0025.38.bw	H3K4me1_CEMT0025.19.bw
Histone	H3K4me1	CEMT0026	H3K4me1_CEMT0026.38.bw	H3K4me1_CEMT0026.19.bw
Histone	H3K4me1	CEMT0027	H3K4me1_CEMT0027.38.bw	H3K4me1_CEMT0027.19.bw
Histone	H3K4me1	CEMT0028	H3K4me1_CEMT0028.38.bw	H3K4me1_CEMT0028.19.bw
Histone	H3K4me1	CEMT0029	H3K4me1_CEMT0029.38.bw	H3K4me1_CEMT0029.19.bw
Histone	H3K4me1	CEMT0030	H3K4me1_CEMT0030.38.bw	H3K4me1_CEMT0030.19.bw
Histone	H3K4me1	CEMT0031	H3K4me1_CEMT0031.38.bw	H3K4me1_CEMT0031.19.bw
Histone	H3K4me1	CEMT0032	H3K4me1_CEMT0032.38.bw	H3K4me1_CEMT0032.19.bw
Histone	H3K4me1	CEMT0033	H3K4me1_CEMT0033.38.bw	H3K4me1_CEMT0033.19.bw
Histone	H3K4me1	CEMT0034	H3K4me1_CEMT0034.38.bw	H3K4me1_CEMT0034.19.bw
Histone	H3K4me1	CEMT0035	H3K4me1_CEMT0035.38.bw	H3K4me1_CEMT0035.19.bw
Histone	H3K4me1	CEMT0036	H3K4me1_CEMT0036.38.bw	H3K4me1_CEMT0036.19.bw
Histone	H3K4me1	CEMT0037	H3K4me1_CEMT0037.38.bw	H3K4me1_CEMT0037.19.bw
Histone	H3K4me1	CEMT0038	H3K4me1_CEMT0038.38.bw	H3K4me1_CEMT0038.19.bw
Histone	H3K4me1	CEMT0047	H3K4me1_CEMT0047.38.bw	H3K4me1_CEMT0047.19.bw
Histone	H3K4me1	CEMT0050	H3K4me1_CEMT0050.38.bw	H3K4me1_CEMT0050.19.bw
Histone	H3K4me1	CEMT0051	H3K4me1_CEMT0051.38.bw	H3K4me1_CEMT0051.19.bw
Histone	H3K4me1	CEMT0052	H3K4me1_CEMT0052.38.bw	H3K4me1_CEMT0052.19.bw
Histone	H3K4me1	CEMT0053	H3K4me1_CEMT0053.38.bw	H3K4me1_CEMT0053.19.bw
Histone	H3K4me1	CEMT0054	H3K4me1_CEMT0054.38.bw	H3K4me1_CEMT0054.19.bw
Histone	H3K4me1	CEMT0055	H3K4me1_CEMT0055.38.bw	H3K4me1_CEMT0055.19.bw
Histone	H3K4me1	CEMT0056	H3K4me1_CEMT0056.38.bw	H3K4me1_CEMT0056.19.bw
Histone	H3K4me1	CEMT0057	H3K4me1_CEMT0057.38.bw	H3K4me1_CEMT0057.19.bw
Histone	H3K4me1	CEMT0058	H3K4me1_CEMT0058.38.bw	H3K4me1_CEMT0058.19.bw
Histone	H3K4me1	CEMT0059	H3K4me1_CEMT0059.38.bw	H3K4me1_CEMT0059.19.bw
Histone	H3K4me1	CEMT0060	H3K4me1_CEMT0060.38.bw	H3K4me1_CEMT0060.19.bw
Histone	H3K4me1	CEMT0061	H3K4me1_CEMT0061.38.bw	H3K4me1_CEMT0061.19.bw
Histone	H3K4me1	CEMT0062	H3K4me1_CEMT0062.38.bw	H3K4me1_CEMT0062.19.bw
Histone	H3K4me1	CEMT0063	H3K4me1_CEMT0063.38.bw	H3K4me1_CEMT0063.19.bw
Histone	H3K4me1	CEMT0064	H3K4me1_CEMT0064.38.bw	H3K4me1_CEMT0064.19.bw
Histone	H3K4me1	CEMT0065	H3K4me1_CEMT0065.38.bw	H3K4me1_CEMT0065.19.bw
Histone	H3K4me1	CEMT0066	H3K4me1_CEMT0066.38.bw	H3K4me1_CEMT0066.19.bw
Histone	H3K4me1	CEMT0067	H3K4me1_CEMT0067.38.bw	H3K4me1_CEMT0067.19.bw
Histone	H3K4me1	CEMT0068	H3K4me1_CEMT0068.38.bw	H3K4me1_CEMT0068.19.bw
Histone	H3K4me1	CEMT0072	H3K4me1_CEMT0072.38.bw	H3K4me1_CEMT0072.19.bw
Histone	H3K4me1	CEMT0073	H3K4me1_CEMT0073.38.bw	H3K4me1_CEMT0073.19.bw
Histone	H3K4me1	CEMT0074	H3K4me1_CEMT0074.38.bw	H3K4me1_CEMT0074.19.bw
Histone	H3K4me1	CEMT0075	H3K4me1_CEMT0075.38.bw	H3K4me1_CEMT0075.19.bw

Histone	H3K4me1	CEMT0076	H3K4me1_CEMT0076.38.bw	H3K4me1_CEMT0076.19.bw
Histone	H3K4me1	CEMT0078	H3K4me1_CEMT0078.38.bw	H3K4me1_CEMT0078.19.bw
Histone	H3K4me1	CEMT0079	H3K4me1_CEMT0079.38.bw	H3K4me1_CEMT0079.19.bw
Histone	H3K4me1	CEMT0081	H3K4me1_CEMT0081.38.bw	H3K4me1_CEMT0081.19.bw
Histone	H3K4me1	CEMT0094	H3K4me1_CEMT0094.38.bw	H3K4me1_CEMT0094.19.bw
Histone	H3K4me1	CEMT0095	H3K4me1_CEMT0095.38.bw	H3K4me1_CEMT0095.19.bw
Histone	H3K4me1	CEMT0096	H3K4me1_CEMT0096.38.bw	H3K4me1_CEMT0096.19.bw
Histone	H3K4me1	CEMT0097	H3K4me1_CEMT0097.38.bw	H3K4me1_CEMT0097.19.bw
Histone	H3K4me1	CEMT0149	H3K4me1_CEMT0149.38.bw	H3K4me1_CEMT0149.19.bw
Histone	H3K4me1	CEMT0150	H3K4me1_CEMT0150.38.bw	H3K4me1_CEMT0150.19.bw
Histone	H3K4me3	CEMT0004	H3K4me3_CEMT0004.38.bw	H3K4me3_CEMT0004.19.bw
Histone	H3K4me3	CEMT0005	H3K4me3_CEMT0005.38.bw	H3K4me3_CEMT0005.19.bw
Histone	H3K4me3	CEMT0006	H3K4me3_CEMT0006.38.bw	H3K4me3_CEMT0006.19.bw
Histone	H3K4me3	CEMT0007	H3K4me3_CEMT0007.38.bw	H3K4me3_CEMT0007.19.bw
Histone	H3K4me3	CEMT0008	H3K4me3_CEMT0008.38.bw	H3K4me3_CEMT0008.19.bw
Histone	H3K4me3	CEMT0009	H3K4me3_CEMT0009.38.bw	H3K4me3_CEMT0009.19.bw
Histone	H3K4me3	CEMT0019	H3K4me3_CEMT0019.38.bw	H3K4me3_CEMT0019.19.bw
Histone	H3K4me3	CEMT0021	H3K4me3_CEMT0021.38.bw	H3K4me3_CEMT0021.19.bw
Histone	H3K4me3	CEMT0022	H3K4me3_CEMT0022.38.bw	H3K4me3_CEMT0022.19.bw
Histone	H3K4me3	CEMT0023	H3K4me3_CEMT0023.38.bw	H3K4me3_CEMT0023.19.bw
Histone	H3K4me3	CEMT0025	H3K4me3_CEMT0025.38.bw	H3K4me3_CEMT0025.19.bw
Histone	H3K4me3	CEMT0026	H3K4me3_CEMT0026.38.bw	H3K4me3_CEMT0026.19.bw
Histone	H3K4me3	CEMT0027	H3K4me3_CEMT0027.38.bw	H3K4me3_CEMT0027.19.bw
Histone	H3K4me3	CEMT0028	H3K4me3_CEMT0028.38.bw	H3K4me3_CEMT0028.19.bw
Histone	H3K4me3	CEMT0029	H3K4me3_CEMT0029.38.bw	H3K4me3_CEMT0029.19.bw
Histone	H3K4me3	CEMT0030	H3K4me3_CEMT0030.38.bw	H3K4me3_CEMT0030.19.bw
Histone	H3K4me3	CEMT0031	H3K4me3_CEMT0031.38.bw	H3K4me3_CEMT0031.19.bw
Histone	H3K4me3	CEMT0032	H3K4me3_CEMT0032.38.bw	H3K4me3_CEMT0032.19.bw
Histone	H3K4me3	CEMT0033	H3K4me3_CEMT0033.38.bw	H3K4me3_CEMT0033.19.bw
Histone	H3K4me3	CEMT0034	H3K4me3_CEMT0034.38.bw	H3K4me3_CEMT0034.19.bw
Histone	H3K4me3	CEMT0035	H3K4me3_CEMT0035.38.bw	H3K4me3_CEMT0035.19.bw
Histone	H3K4me3	CEMT0036	H3K4me3_CEMT0036.38.bw	H3K4me3_CEMT0036.19.bw
Histone	H3K4me3	CEMT0037	H3K4me3_CEMT0037.38.bw	H3K4me3_CEMT0037.19.bw
Histone	H3K4me3	CEMT0038	H3K4me3_CEMT0038.38.bw	H3K4me3_CEMT0038.19.bw
Histone	H3K4me3	CEMT0047	H3K4me3_CEMT0047.38.bw	H3K4me3_CEMT0047.19.bw
Histone	H3K4me3	CEMT0050	H3K4me3_CEMT0050.38.bw	H3K4me3_CEMT0050.19.bw
Histone	H3K4me3	CEMT0051	H3K4me3_CEMT0051.38.bw	H3K4me3_CEMT0051.19.bw
Histone	H3K4me3	CEMT0052	H3K4me3_CEMT0052.38.bw	H3K4me3_CEMT0052.19.bw
Histone	H3K4me3	CEMT0053	H3K4me3_CEMT0053.38.bw	H3K4me3_CEMT0053.19.bw
Histone	H3K4me3	CEMT0054	H3K4me3_CEMT0054.38.bw	H3K4me3_CEMT0054.19.bw
Histone	H3K4me3	CEMT0055	H3K4me3_CEMT0055.38.bw	H3K4me3_CEMT0055.19.bw
Histone	H3K4me3	CEMT0056	H3K4me3_CEMT0056.38.bw	H3K4me3_CEMT0056.19.bw

Histone	H3K4me3	CEMT0057	H3K4me3_CEMT0057.38.bw	H3K4me3_CEMT0057.19.bw
Histone	H3K4me3	CEMT0058	H3K4me3_CEMT0058.38.bw	H3K4me3_CEMT0058.19.bw
Histone	H3K4me3	CEMT0059	H3K4me3_CEMT0059.38.bw	H3K4me3_CEMT0059.19.bw
Histone	H3K4me3	CEMT0060	H3K4me3_CEMT0060.38.bw	H3K4me3_CEMT0060.19.bw
Histone	H3K4me3	CEMT0061	H3K4me3_CEMT0061.38.bw	H3K4me3_CEMT0061.19.bw
Histone	H3K4me3	CEMT0062	H3K4me3_CEMT0062.38.bw	H3K4me3_CEMT0062.19.bw
Histone	H3K4me3	CEMT0063	H3K4me3_CEMT0063.38.bw	H3K4me3_CEMT0063.19.bw
Histone	H3K4me3	CEMT0064	H3K4me3_CEMT0064.38.bw	H3K4me3_CEMT0064.19.bw
Histone	H3K4me3	CEMT0065	H3K4me3_CEMT0065.38.bw	H3K4me3_CEMT0065.19.bw
Histone	H3K4me3	CEMT0066	H3K4me3_CEMT0066.38.bw	H3K4me3_CEMT0066.19.bw
Histone	H3K4me3	CEMT0067	H3K4me3_CEMT0067.38.bw	H3K4me3_CEMT0067.19.bw
Histone	H3K4me3	CEMT0068	H3K4me3_CEMT0068.38.bw	H3K4me3_CEMT0068.19.bw
Histone	H3K4me3	CEMT0072	H3K4me3_CEMT0072.38.bw	H3K4me3_CEMT0072.19.bw
Histone	H3K4me3	CEMT0073	H3K4me3_CEMT0073.38.bw	H3K4me3_CEMT0073.19.bw
Histone	H3K4me3	CEMT0074	H3K4me3_CEMT0074.38.bw	H3K4me3_CEMT0074.19.bw
Histone	H3K4me3	CEMT0075	H3K4me3_CEMT0075.38.bw	H3K4me3_CEMT0075.19.bw
Histone	H3K4me3	CEMT0076	H3K4me3_CEMT0076.38.bw	H3K4me3_CEMT0076.19.bw
Histone	H3K4me3	CEMT0078	H3K4me3_CEMT0078.38.bw	H3K4me3_CEMT0078.19.bw
Histone	H3K4me3	CEMT0079	H3K4me3_CEMT0079.38.bw	H3K4me3_CEMT0079.19.bw
Histone	H3K4me3	CEMT0081	H3K4me3_CEMT0081.38.bw	H3K4me3_CEMT0081.19.bw
Histone	H3K4me3	CEMT0094	H3K4me3_CEMT0094.38.bw	H3K4me3_CEMT0094.19.bw
Histone	H3K4me3	CEMT0095	H3K4me3_CEMT0095.38.bw	H3K4me3_CEMT0095.19.bw
Histone	H3K4me3	CEMT0096	H3K4me3_CEMT0096.38.bw	H3K4me3_CEMT0096.19.bw
Histone	H3K4me3	CEMT0097	H3K4me3_CEMT0097.38.bw	H3K4me3_CEMT0097.19.bw
Histone	H3K4me3	CEMT0149	H3K4me3_CEMT0149.38.bw	H3K4me3_CEMT0149.19.bw
Histone	H3K4me3	CEMT0150	H3K4me3_CEMT0150.38.bw	H3K4me3_CEMT0150.19.bw
Histone	H3K9me3	CEMT0004	H3K9me3_CEMT0004.38.bw	H3K9me3_CEMT0004.19.bw
Histone	H3K9me3	CEMT0005	H3K9me3_CEMT0005.38.bw	H3K9me3_CEMT0005.19.bw
Histone	H3K9me3	CEMT0006	H3K9me3_CEMT0006.38.bw	H3K9me3_CEMT0006.19.bw
Histone	H3K9me3	CEMT0007	H3K9me3_CEMT0007.38.bw	H3K9me3_CEMT0007.19.bw
Histone	H3K9me3	CEMT0008	H3K9me3_CEMT0008.38.bw	H3K9me3_CEMT0008.19.bw
Histone	H3K9me3	CEMT0009	H3K9me3_CEMT0009.38.bw	H3K9me3_CEMT0009.19.bw
Histone	H3K9me3	CEMT0019	H3K9me3_CEMT0019.38.bw	H3K9me3_CEMT0019.19.bw
Histone	H3K9me3	CEMT0021	H3K9me3_CEMT0021.38.bw	H3K9me3_CEMT0021.19.bw
Histone	H3K9me3	CEMT0022	H3K9me3_CEMT0022.38.bw	H3K9me3_CEMT0022.19.bw
Histone	H3K9me3	CEMT0023	H3K9me3_CEMT0023.38.bw	H3K9me3_CEMT0023.19.bw
Histone	H3K9me3	CEMT0025	H3K9me3_CEMT0025.38.bw	H3K9me3_CEMT0025.19.bw
Histone	H3K9me3	CEMT0026	H3K9me3_CEMT0026.38.bw	H3K9me3_CEMT0026.19.bw
Histone	H3K9me3	CEMT0027	H3K9me3_CEMT0027.38.bw	H3K9me3_CEMT0027.19.bw
Histone	H3K9me3	CEMT0028	H3K9me3_CEMT0028.38.bw	H3K9me3_CEMT0028.19.bw
Histone	H3K9me3	CEMT0029	H3K9me3_CEMT0029.38.bw	H3K9me3_CEMT0029.19.bw
Histone	H3K9me3	CEMT0030	H3K9me3_CEMT0030.38.bw	H3K9me3_CEMT0030.19.bw

Histone	H3K9me3	CEMT0031	H3K9me3_CEMT0031.38.bw	H3K9me3_CEMT0031.19.bw
Histone	H3K9me3	CEMT0032	H3K9me3_CEMT0032.38.bw	H3K9me3_CEMT0032.19.bw
Histone	H3K9me3	CEMT0033	H3K9me3_CEMT0033.38.bw	H3K9me3_CEMT0033.19.bw
Histone	H3K9me3	CEMT0034	H3K9me3_CEMT0034.38.bw	H3K9me3_CEMT0034.19.bw
Histone	H3K9me3	CEMT0035	H3K9me3_CEMT0035.38.bw	H3K9me3_CEMT0035.19.bw
Histone	H3K9me3	CEMT0036	H3K9me3_CEMT0036.38.bw	H3K9me3_CEMT0036.19.bw
Histone	H3K9me3	CEMT0037	H3K9me3_CEMT0037.38.bw	H3K9me3_CEMT0037.19.bw
Histone	H3K9me3	CEMT0038	H3K9me3_CEMT0038.38.bw	H3K9me3_CEMT0038.19.bw
Histone	H3K9me3	CEMT0047	H3K9me3_CEMT0047.38.bw	H3K9me3_CEMT0047.19.bw
Histone	H3K9me3	CEMT0050	H3K9me3_CEMT0050.38.bw	H3K9me3_CEMT0050.19.bw
Histone	H3K9me3	CEMT0051	H3K9me3_CEMT0051.38.bw	H3K9me3_CEMT0051.19.bw
Histone	H3K9me3	CEMT0052	H3K9me3_CEMT0052.38.bw	H3K9me3_CEMT0052.19.bw
Histone	H3K9me3	CEMT0053	H3K9me3_CEMT0053.38.bw	H3K9me3_CEMT0053.19.bw
Histone	H3K9me3	CEMT0054	H3K9me3_CEMT0054.38.bw	H3K9me3_CEMT0054.19.bw
Histone	H3K9me3	CEMT0055	H3K9me3_CEMT0055.38.bw	H3K9me3_CEMT0055.19.bw
Histone	H3K9me3	CEMT0056	H3K9me3_CEMT0056.38.bw	H3K9me3_CEMT0056.19.bw
Histone	H3K9me3	CEMT0057	H3K9me3_CEMT0057.38.bw	H3K9me3_CEMT0057.19.bw
Histone	H3K9me3	CEMT0058	H3K9me3_CEMT0058.38.bw	H3K9me3_CEMT0058.19.bw
Histone	H3K9me3	CEMT0059	H3K9me3_CEMT0059.38.bw	H3K9me3_CEMT0059.19.bw
Histone	H3K9me3	CEMT0060	H3K9me3_CEMT0060.38.bw	H3K9me3_CEMT0060.19.bw
Histone	H3K9me3	CEMT0061	H3K9me3_CEMT0061.38.bw	H3K9me3_CEMT0061.19.bw
Histone	H3K9me3	CEMT0062	H3K9me3_CEMT0062.38.bw	H3K9me3_CEMT0062.19.bw
Histone	H3K9me3	CEMT0063	H3K9me3_CEMT0063.38.bw	H3K9me3_CEMT0063.19.bw
Histone	H3K9me3	CEMT0064	H3K9me3_CEMT0064.38.bw	H3K9me3_CEMT0064.19.bw
Histone	H3K9me3	CEMT0065	H3K9me3_CEMT0065.38.bw	H3K9me3_CEMT0065.19.bw
Histone	H3K9me3	CEMT0066	H3K9me3_CEMT0066.38.bw	H3K9me3_CEMT0066.19.bw
Histone	H3K9me3	CEMT0067	H3K9me3_CEMT0067.38.bw	H3K9me3_CEMT0067.19.bw
Histone	H3K9me3	CEMT0068	H3K9me3_CEMT0068.38.bw	H3K9me3_CEMT0068.19.bw
Histone	H3K9me3	CEMT0072	H3K9me3_CEMT0072.38.bw	H3K9me3_CEMT0072.19.bw
Histone	H3K9me3	CEMT0073	H3K9me3_CEMT0073.38.bw	H3K9me3_CEMT0073.19.bw
Histone	H3K9me3	CEMT0074	H3K9me3_CEMT0074.38.bw	H3K9me3_CEMT0074.19.bw
Histone	H3K9me3	CEMT0075	H3K9me3_CEMT0075.38.bw	H3K9me3_CEMT0075.19.bw
Histone	H3K9me3	CEMT0076	H3K9me3_CEMT0076.38.bw	H3K9me3_CEMT0076.19.bw
Histone	H3K9me3	CEMT0078	H3K9me3_CEMT0078.38.bw	H3K9me3_CEMT0078.19.bw
Histone	H3K9me3	CEMT0079	H3K9me3_CEMT0079.38.bw	H3K9me3_CEMT0079.19.bw
Histone	H3K9me3	CEMT0081	H3K9me3_CEMT0081.38.bw	H3K9me3_CEMT0081.19.bw
Histone	H3K9me3	CEMT0094	H3K9me3_CEMT0094.38.bw	H3K9me3_CEMT0094.19.bw
Histone	H3K9me3	CEMT0095	H3K9me3_CEMT0095.38.bw	H3K9me3_CEMT0095.19.bw
Histone	H3K9me3	CEMT0096	H3K9me3_CEMT0096.38.bw	H3K9me3_CEMT0096.19.bw
Histone	H3K9me3	CEMT0097	H3K9me3_CEMT0097.38.bw	H3K9me3_CEMT0097.19.bw
Histone	H3K9me3	CEMT0149	H3K9me3_CEMT0149.38.bw	H3K9me3_CEMT0149.19.bw
Histone	H3K9me3	CEMT0150	H3K9me3_CEMT0150.38.bw	H3K9me3_CEMT0150.19.bw

Transcription factor	AR		AR.38.bw	AR.19.bw
Transcription factor	ARID1B		ARID1B.38.bw	ARID1B.19.bw
Transcription factor	BACH1		BACH1.38.bw	BACH1.19.bw
Transcription factor	BCOR		BCOR.38.bw	BCOR.19.bw
Transcription factor	BMI1		BMI1.38.bw	BMI1.19.bw
Transcription factor	BRCA1		BRCA1.38.bw	BRCA1.19.bw
Transcription factor	CDK9		CDK9.38.bw	CDK9.19.bw
Transcription factor	CTCF		CTCF.38.bw	CTCF.19.bw
Transcription factor	ELF1		ELF1.38.bw	ELF1.19.bw
Transcription factor	GATA3		GATA3.38.bw	GATA3.19.bw
Transcription factor	NFATC1		NFATC1.38.bw	NFATC1.19.bw
Transcription factor	OTX2		OTX2.38.bw	OTX2.19.bw
Transcription factor	SOX10		SOX10.38.bw	SOX10.19.bw
Transcription factor	SOX13		SOX13.38.bw	SOX13.19.bw
Transcription factor	SOX2		SOX2.38.bw	SOX2.19.bw
Transcription factor	TP53		TP53.38.bw	TP53.19.bw
Transcription factor	ZNF175		ZNF175.38.bw	ZNF175.19.bw
Transcription factor	ESR1		ESR1.38.bw	ESR1.19.bw

Supplementary Table 4. Enrichment odds ratios for duplication and not CG in full CpGs dataset

**Region**	**Odds ratio**		
	**Duplication**	**Alternative chromosome**	**Not CG**
CpG islands	0.325921	0.765058	0.324116
CpG shelves	1.203549	1.289100	1.248004
CpG shores	0.856068	0.931636	0.858670
Blacklist	3.541094	**20.277765**	4.145776
Gapped-in-both	**64.125956**	**28.088276**	**49.615954**
Gapped-in-hg19	**59.441170**	**85.332853**	**45.161457**
Gapped-in-hg38	**181.946429**	1.000000	**181.946429**
Repeat	1.009862	0.992588	1.007631

SNP150common	6.030971	0.846301	6.054714
Ungapped	0.987189	0.001046	0.988539
3UTRs	0.646515	0.082226	0.796090
5UTRs	0.363641	0.064656	0.365859
Cds	0.632957	0.274981	0.603096
Exon-intron boundaries	0.951297	0.767411	1.012693
Exons	0.743113	0.767212	0.751581
First exons	0.568685	0.591585	0.567588
Intergenic	1.148251	0.644222	1.151976
Intron-exon boundaries	1.110391	0.917266	1.181289
Introns	1.242316	1.257731	1.253412
Promoters	0.583792	1.012914	0.635340

Supplementary Table 5. Enrichment odds ratios for delta ≥0.20 in sample CEMT0062_A59692 and median values among 43 WGBS samples

**Region**	**Odds ratio**	
	**CEMT0062_A59692**	**43 samples**
CpG islands	1.338679	0.923564
CpG shelves	1.093735	1.141845
CpG shores	1.143677	1.091264
Blacklist	**33.693798**	**30.119921**
Gapped-in-both	**16.895092**	**10.517039**
Gapped-in-hg19	**19.741464**	**12.036589**
Gapped-in-hg38	1.999976	1.000000
Repeat	1.155897	1.190682
SNP150common	1.278573	1.445347
Ungapped	0.994035	0.995153
3UTRs	0.341806	0.539204
5UTRs	0.193906	0.220743
Cds	0.357448	0.394504
Exon-intron boundaries	0.426241	0.482830
Exons	0.422516	0.484408
First exons	0.441777	0.413011
Intergenic	1.367483	1.326373
Intron-exon boundaries	0.418266	0.503968
Introns	0.636292	0.691399
Promoters	0.726143	0.611822

Supplementary Table 6. Medians of enrichment odds ratios for loss and gain intervals among 366 ChIP-Seq samples, comparison of *UCSC liftOver* and *segment_liftover*

**Region**	**Odds ratio**	
	UCSC	***segment***
CpG islands	1.890110	1.905455
CpG shelves	1.665683	1.655707
CpG shores	1.744103	1.724479

Blacklist	4.911317	4.267090
Gapped-in-both	1.627196	1.620127
Gapped-in-hg19	2.874979	2.784645
Gapped-in-hg38	3.235616	2.917922
Repeat	0.966708	0.962967
SNP150common	1.149601	1.103458
Ungapped	1.045007	1.046584
3UTRs	1.289819	1.262987
5UTRs	1.613840	1.524786
Cds	1.348518	1.375390
Exon-intron boundaries	1.411851	1.416393
Exons	1.420311	1.438132
First exons	1.603256	1.590239
Intergenic	0.792718	0.778285
Intron-exon boundaries	1.373464	1.417218
Introns	1.269082	1.250719
Promoters	1.607443	1.614506
